# Phytonutritional Composition and Antioxidant Properties of Southern African, Purple-Fleshed Sweet Potato (*Ipomoea batatas* (L.) Lam.) Storage Roots

**DOI:** 10.3390/antiox13030338

**Published:** 2024-03-11

**Authors:** Ayanda Ngcobo, Sephora Mutombo Mianda, Faith Seke, Laurie M. Sunette, Dharini Sivakumar

**Affiliations:** 1 Department of Crop Sciences, Tshwane University of Technology, Pretoria 0183, South Africa; ayandangcobo36@gmail.com (A.N.); miandamutombos@tut.ac.za (S.M.M.); sekef@tut.ac.za (F.S.); 2 Agricultural Research Council-Vegetable, Industrial and Medicinal Plants, Private Bag X293, Pretoria 0001, South Africa; slaurie@arc.agric.za; 3 Centre for Nutrition and Food Sciences, Queensland Alliance for Agriculture and Food Innovation, Indooroopilly, QLD 4068, Australia

**Keywords:** morphological traits, caffeoylquinic acid derivative, cyanidin glycosides, peonidin glycosides, ferric reducing antioxidant power

## Abstract

Purple sweet potatoes (*Ipomoea batatas* (L.) genotype) in Southern Africa have a phytonutritional composition and antioxidant properties that can increase incomes and improve nutrition. This study compared the phytonutrient composition and antioxidant properties of four purple-colour sweet potato genotypes (local Purple-purple, ‘2019-1-1’, and USA genotypes, ‘08-21P’ and ‘16-283P’). These purple sweet potato genotypes were characterised by UPLC/QTOF/MS and 16 phenolic compounds were identified. Purple-purple (very dark purple) showed the highest concentration of cyanidins and peonidin derivatives. Chlorogenic acid derivatives were highest in the genotype ‘16-283P’. ‘Puple-purple’ and ‘16-283P’ displayed the strongest antioxidant power and scavenging activities. Diaffeoylquinic acid isomer 1 was identified as the marker candidate for distinguishing the four purple sweet potato genotypes. Southern Africa’s highest-protein sweet potato genotypes are Purple-purple (28.81 g/100 g) and ‘08-21 P’ (24.31 g/100 g). A 13.65 g portion of ‘2019-1-1′ would meet the Recommended Dietary Allowance (RDA) for iron for men, while 25.59 g would meet the RDA for children, and 30.72 g would meet the RDA for women. The sweet potato root of genotype ‘2019-1-1′ provides 31.43 g of Zn per day for children and 22.86 g for adults. The roots of local cultivar Purple-purple can be used as functional food ingredients.

## 1. Introduction

Sweet potatoes are dicotyledonous plants (*Ipomoea batatas* (L.) Lam) of the *Convolvulaceae* family [1]. It is one of the most widely grown crops in the world and is considered a food security crop. Sweet potato is among several crops that have been used successfully for biofortification to reduce hidden hunger, specifically, a micronutrient deficiency aligned with a shortage of vitamin A, by breeding orange-fleshed cultivars rich in carotenoids [2]. Sweet potatoes are key staple food in South America, the Caribbean, Asia, and Africa and are a good source of calcium, iron, zinc, vitamins A and C, magnesium, phosphorus, and potassium [3]. The Food and Agriculture Organisation (FAO) stated that 109 nations produced sweet potatoes in 2019. China produced 46.6 million metric tons of sweet potatoes in 2022 [4]. The production of sweet potatoes increased by 1.5% in 2022 after two years of decline in Africa [5]. Malawi, Nigeria, Tanzania, Uganda, Ethiopia, Angola, Rwanda, Madagascar, Burundi, and Kenya are 10 of the world’s top 20 sweet potato-producing countries (Food and Agriculture Organization) [6]. Sweet potatoes are commonly cultivated for the consumption of their storage roots of various pleasant colours from cream/white, purple, and yellow to orange [7]. There are 131 million tons of sweet potatoes grown worldwide each year, and it ranks third in importance after potatoes [8]. The varieties of staple roots in Sub-Saharan Africa are white- or cream-fleshed, distinguished by having high starch content [9]. 

In South Africa, the Agricultural Research Council programme includes the breeding and commercialisation of sweet potatoes, developing cultivars with a tolerance to major diseases and a better yield and root quality traits, i.e., high levels of anthocyanins [10]. The South African Agricultural Research Council has released 33 genotypes thus far, with some, like ‘Ribbok’, ‘Bosbok’, and ‘Blesbok’, being commercialised. ‘Blesbok’, a cultivar with purple skin, is known for its high yield and low dry matter content [11,12]. Ndou and Monate have a high yield and high dry matter content, with cream flesh and cream skin [13]. Additionally, there are orange-fleshed cultivars with a high β-carotene content, such as Bophelo and Khumo. Among the more recent developments are breeding lines FS10-25 and FS10-21, which produce cream-fleshed storage roots with purple skin known for their excellent storability and wilt tolerance.

Recently, breeders have shown interest in purple-fleshed sweet potato cultivars, as highlighted by Parker et al. [14]. The main aim of breeding is to produce cultivars with high levels of anthocyanin and antioxidant capacity [15]. Purple sweet potatoes are rich in anthocyanins, starch, polysaccharides, caffeoylquinic acid derivatives, vitamins, and minerals [16]. Additionally, anthocyanins have antimutagenic, hepato-protective, antihypertensive, antihyperglycemic, antimicrobial, anti-inflammatory, and anti-obesity properties [17]. According to Ghasemzadeh et al. [18] and Dwiyanti et al. [19], compared to the anthocyanin found in red cabbage, elderberries, blueberries, and red corn, the pigment anthocyanin in purple sweet potatoes has a higher stability. Sweet potatoes with purple flesh are rich in cyanidin and peonidin glycosides, which acylate their sophorose through the presence of p-hydroxybenzoyl, caffeoyl, and feruloyl moieties [20]. However, the content of anthocyanin, caffeoylquinic acid derivatives, protein, fibre, Fe, Zn, and the antioxidant activity of purple varieties grown in South Africa are unknown. The objective of the present study was to investigate phytonutritional composition, including mineral elements Fe and Zn, total and individual phenolic compounds, and the antioxidant activities of the purple-colour flesh sweet potato roots found in the Southern African region—the Agricultural Research Council breeding lines ‘2019-1-1’ (purple skin with purple, cream-ring flesh), the locally collected genotype Purple-purple (dark purple skin with very dark purple flesh), and two imported cultivars from the USA (‘08-21P’ and ‘16-283P’).

## 2. Materials and Methods

### 2.1. Plant Material

**Figure 1:**
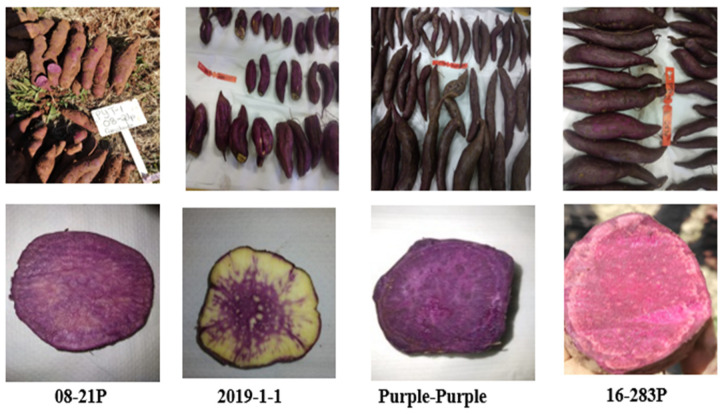
Shape and size of roots and distribution of anthocyanin in four purple-fleshed genotypes.

The locally sourced genotype (Purple-purple), one Agricultural Research Council breeding line (‘2019-1-1′), and two imports from the USA (‘08-21P’ and ‘16-283P’) (Figure 1) were grown during the 2022 and 2023 growing seasons at the Roodeplaat campus (25°36’26” S, 28°33’40” E, altitude 1220 m above sea level) of the Agricultural Research Council–Vegetables, Industrial and Medicinal Plants (ARC-VIMP) located in northern Gauteng, South Africa.

### 2.2. Total Marketable Yield and Morphological Assessment

After harvest, the total marketable yield was determined (Scale Model Alpha 2 LCD, Jiangsu, China) and the results were expressed as kg/20 plants. On the day of harvesting, ten storage roots from each genotype were chosen for morphological assessment.

Diameter (cm), length (cm), and cortex thickness (mm) were measured using a calliper. The shape, pigmentation, and distribution of pigmentation of the storage roots were recorded based on International Potato Centre (CIP) sweet potato descriptors (Huamari, 1992). The pigmentation and distribution of pigmentation of the storage roots were recorded as explained by Selokela et al. [21].

### 2.3. Colour Measurement

Skin and flesh colour were recorded from 10 replicate samples using a chromameter (Model CR-400 Minolta, Konica Minolta Sensing, Inc., Osaka, Japan) and expressed as CIELab* values. The chromameter was calibrated to a white calibration plate. L* (+100 = white, −100 = black), the Hunter colour scale’s tri-stimulus values (L: light, a (+a = red), b (b = blue, +b = yellow, a = green), chromaticity or C* (colour intensity) and hue angle or hº (0 = red, 90 = yellow, 180 = green, 270 = blue) of the flesh [22].

### 2.4. Chemical Analysis

A set of 50 storage roots of each genotype, devoid of deterioration or damage, was selected randomly and pooled, and from those, 10 subsamples were taken for chemical analysis. These were washed with tap water [23] and transported to the Fruit and Vegetable Laboratory at the Tshwane University of Technology, in Pretoria West. Thereafter, the roots were chopped and freeze-dried (VirTis Sp Scientific, Model # 2kBTES-55, Gardiner, NY, USA) at −47 to −53 °C for 72 h and ground into fine powder, then stored at 4 °C until use.

#### 2.4.1. Chemicals

The chemicals and standards mentioned below were purchased from Lasec SA (Pty) Ltd. in Midrand, Gauteng, South Africa.

#### 2.4.2. Total Phenolic Content (TPC)

The TPC was determined from purple-fleshed storage roots using the procedure explained by Hong et al. [24]. Each sample weighing 10 mg was extracted with 10 mL of 80% methanol using magnetic stirring (Edison, H4000-HSB, Sayreville NJ 08872, USA). The TPC was calculated using the standard curve generated with chlorogenic acid, which had concentrations ranging from 0–100 ppm, and the measurements were expressed in milligrams per gram of chlorogenic acid equivalent.

#### 2.4.3. Ferric Reducing Antioxidant Power (FRAP)

The FRAP value was established according to the protocol described by Selokela et al. [21]. The absorbance was then measured at 593 nm using a spectrophotometer. A standard curve was created with standards ranging from 0 to 700 concentrations, and the results were expressed as mM TEAC/g. The equation for the standard curve was Y = 0.0005x + 0.1216, with an R^2^ value of 0.98.

#### 2.4.4. 2,2-Diphenyl-1-picrylhydrazyl (DPPH) Assay

The DPPH scavenging ability was used to measure inhibiting activity with some minor changes, following the method described by Suárez et al. [25]. The inhibiting activity of the storage roots was measured by the decrease in absorbance in the methanol solution of DPPH. The IC₅₀ (mg/mL) was calculated using the concentration versus inhibition % graph.

#### 2.4.5. 2,2′-Azino-bis (3-Ethylbenzothiazoline-6-sulfonic Acid) Scavenging Activity

ABTS scavenging activity was carried out, following the method explained by Seke et al. [26] A measurement was made of the decline in absorbance at 734 nm. A graph of the percentage of inhibition versus the concentration was used to determine the IC_50_ (mg/mL).

### 2.5. Mineral Composition

Fe, Zn, and K were determined using the digest solution in an aliquot and (ICP-OES) inductively coupled plasma optical emission spectrophotometer [(Agilent 725 Series) Santa Clara, CA, USA] device. Mineral content was expressed on a dry weight basis [21].

### 2.6. Quantification of Phenolic Compounds Using UPLC-QTOF/MS

The identification and quantification of predominant phenolic acids and flavonoids were achieved using a UPLC-QTOF/MS system (Waters, Milford, MA, USA) equipped with a Quadrupole 120 time-of-flight (QTOF) mass spectrometer, following the method described by Managa et al. [27] without any modifications. The calibration curve set up using a chlorogenic acid standard was used to quantitatively and semi-quantitatively measure all identified compounds. The content of phenolic compounds in the study is expressed as mg/kg. Data were processed using TargetLynx software as previously reported [28]. The data generated by the UPLC-Q-TOF/MS and HPLC-DAD (individual anthocyanins) analysis were analysed using principal component analysis (PCA) and Projections to Latent Structures–Discriminant Analysis (PLS-DA) approaches to identify the differences between the phenolic profiles of the different genotypes of the sweet potatoes’ roots. The regression equation, and retention times’ limit of detection (LOD) and limit of quantification (LOQ), for anthocyanins and phenolic compounds are shown in Supplementary Table S1.

### 2.7. Proximate Composition

Analyses were conducted to determine protein, total dietary fibre, and fat, following the procedures outlined by the Association of Official Analytical Chemists [29].

### 2.8. Statistical Analysis

Variance was analysed on all data collected and means separation using Tukey’s truly significant difference (Tukey’s HSD). All the data collected in this study were analysed using statistical package design Gen stat 18.1. The data were obtained during the 2022 and 2023 growing seasons. The results of the two harvests were compared, and as the data did not differ, it was pooled together and subjected to analysis of variance (ANOVA) using GenStat 11.1.

## 3. Results and Discussion

### 3.1. Morphological Characteristics of Five Purple-Fleshed Genotypes

The storage roots of different coloured sweet potato genotypes differ greatly from one another in terms of their morphological characteristics (Table 1). Most of the roots were long, elliptical, obovate, ovate, or round. The size and quantity of sweet potato storage roots depend on the root system and other characteristics of the plant, which are influenced by the environmental conditions of the growing areas [30]. Additionally, several factors can affect sweet potato morphology, including the season, agricultural practices, and the characteristics of the plant itself [31]. The skin colour of the storage roots of ‘2019-1-1′, ‘08-21P’, ‘16-283P’, and Purple-purple ranged from light purple to dark purple. The flesh of ‘2019-1-1’ had purple inner rings surrounded by cream outer rings. The roots of ‘08-21P’ were violet and pink-cream, and the roots of ‘16-283P’ were dark purple with a slight ring. Only the roots of Purple-purple showed a very dark purple flesh colour (Figure 1). The surface defects of sweet potato roots ranged from being absent to long thick veins, severe cracks, and alligator-like skin.

Furthermore, the yield of these four genotypes varied significantly during the growing seasons (Table 2). Harvested yields for these four purple sweet potatoes were as follows: ‘08-21P’ produced 20.64 kg/20 plants; ‘2019-1-1’ (5.45 kg/20 plants); Purple-purple (15.48 kg/20 plants); and ‘16-283P’ (7.56 kg/20 plants). The ARC-developed genotype ‘2019-1-1’ showed lower yields than the locally found Purple-purple. The root lengths of these four purple sweet potatoes were 21.80 cm, 20.40 cm, 17.00 cm, and 16.85 cm in the ‘08-21P’, Purple-purple, ‘16-283P’, and ‘2019-1-1′ genotypes, respectively (Table 2). The diameter of the roots varied among the four genotypes. The highest root diameter was observed in ‘2019-1-1’ (5.48), followed by ‘08-21P’ (5.07), Purple-purple (4.26 cm), and ‘16-283P’ (4.18 cm). The Purple-purple (3.9 cm) and ‘08-21P’ (3.40 cm) genotypes showed the largest cortex diameters and were followed by ‘16-283P’ (2.80 cm) and ‘2019-1-1’ (1.16 cm) as shown in Table 2. Sweet potato storage roots are reported to vary in length and diameter [3;303]. The farmer’s choice of sweet potato variety depends on the independent variable weight, since it directly affects crop yield [32]. Furthermore, sweet potato morphology is crucial when screening new genotypes because consumers may reject roots with undesirable traits [32].

### 3.2. Colour Properties

Food colour is a crucial quality parameter for sweet potatoes. Table 3 displays the colour attributes of four storage roots of purple-fleshed sweet potatoes. Purple-purple had the lowest L* value (deep purple) and exhibited a darker colour. Hence, the brightness or darkness of the flesh colour of the roots is determined by the L* value. Light purple flesh colours were seen with a higher L* value, while dark purple flesh colours were seen with a lower L* value [17]. It is also important to note that the proportion of lightness (L*) and colour coordinates a* and b* influence flesh colour. Meanwhile, a positive a* colour coordinate relates to a higher red-colour intensity, while a positive b* colour coordinate relates to a higher yellow intensity. The genotype ‘2019-1-1’ exhibited a higher L* value when compared to Purple-purple and the two genotypes from the USA. However, its a* colour coordinate was similar to that of ‘16-283P’ but lower than both ‘08-21P’ and Purple-purple. The chroma value of genotype ‘2019-1-1’ was higher than ‘08-21P’ but lower than ‘16-283P’ and similar to Purple-purple, indicating a purple and cream flesh colour. In addition, colour values are very important to breeders to breed new varieties (Nakagawa et al. [33]). Genotypes with a deep purple colour are appropriate as constituents for flour colourants and snacks [17]. Sweet potatoes are purple because of anthocyanins. Sweet potatoes contain peonidin and cyanidin, which are the most abundant anthocyanins. The flesh appears red-purple when peonidin levels exceed 1, while it appears purple-blue or grey when cyanidin levels dominate. The genotype ‘08-21P’ consistently had the highest value for the a* colour coordinate in both seasons, while the genotypes ‘2019-1-1′ and ‘16-283P’ had the lowest.

### 3.3. Total Phenolic Content

A comparison of the phenol content of the four genotypes of purple sweet potato roots is shown in Table 4. The ARC-developed genotype ‘2019 1-1’ (50.69 mg/g on dry weight (dw) basis) and USA genotype ‘16-283P’ (53.24 mg/g dw) showed the highest TPC. In contrast, Purple-purple (42.45 mg/g) and ‘08-21′P (43.54 mg/g dw) had the lowest TPC in the growing season. In five Korean purple sweet potato genotypes (‘Sinjami’, ‘Jami’, ‘Yeonjami’, ‘Danjami’, and ‘Borami’), the TPC ranged from 1.80 to 7.37 mg GAE/g DW [34]. In contrast to the authors, our values were much higher than those of all the Korean purple sweet potato genotypes. This is because they used gallic acid instead of chlorogenic acid, a phenolic compound found in sweet potatoes. The study by Franková et al. [35] found that flesh colour was a major factor influencing polyphenol levels in sweet potatoes. The purple genotype (414-purple) yielded 1.5 and 3.8 times higher polyphenol levels than the other genotypes such as ‘Beauregard’ and ‘O’Henry’ [35]. The results from our study, however, contradict this statement, because the TPC of the genotype ‘2019-1-1‘ (purple and cream) was higher than ‘16-283P’ (purple-with-orange-spots flesh); (D) ‘08-21P’ (purple-with-cream flesh); and (E) Purple-purple (dark purple flesh).

### 3.4. UHPLC-QTOF-MS Identification and Characterisation of Phenolic Compounds

#### 3.4.1. Chlorogenic Acid Derivatives and Flavonoids

The main phenolic compounds in sweet potatoes are chlorogenic acids [36]. In all four genotypes, chlorogenic acid isomers (3-*O*-Caffeoylquinic acid; 3CQA), neochlorogenic acid (5-*O*-caffeoylquinic acid; 5CQA), 1,3-dicaffeoylquinic acid (1,3-diCQA), dicaffeoylquinic acid isomer 1diCQA, dicaffeoylquinic acid isomer 2, diCQA, dicaffeoylquinic acid isomer 3diCQA, 3-O-caffeoyl-4-O-methylquinic acid, 3,5-dicaffeoylquinic methyl ester, and quercetin 3,4’-diglucoside were detected in this study (Table 5). The characterisation and MS spectra of these metabolites are described in Supplementary Figures S1, S3–S11. Out of the four purple sweet potato genotypes, the highest concentration of 3CQA isomer, all diCQA isomers, 3,5-dicaffeoylquinic methyl ester, and quercetin 3,4’-diglucoside was found in the ‘16-283P’ genotype. In contrast, the highest concentrations of 5CQA were found in the genotypes ‘16-283P’ and Purple-purple. In addition, genotypes ‘16-283P’ and ‘08-21P’ had the highest concentrations of diCQA isomer 2. Furthermore, the ‘08-21P’ genotype contained the highest concentration of 1,3-diCQA. On the other hand, the Purple-purple genotype had the highest concentrations of 3CQA isomer and 3-*O*-Caffeoyl-4-*O*-methylquinic acid compared to all the other three genotypes. Genotype ‘08-21P’ had the highest concentration of 1,3-diCQA. Overall, the ‘16-283P’ genotype had the largest proportion of chlorogenic acid derivatives and quercetin 3,4’-diglucoside compared to the other genotypes in this study. It has been observed that sweet potatoes grown in North Italy, specifically the ‘Beauregard’ genotype, have higher concentrations of 3-CQA (205.5 mg kg^−1^ DW) [37]. In Italy, white-fleshed and orange-fleshed sweet potato roots were found to have 3-CQA levels of 436 and 221–333 mg kg^−1^ DW, respectively [38]. On the other hand, a study conducted on four sweet potato genotypes from China showed that the concentration of chlorogenic acids varied between 300–730 mg kg^−1^ DW of 3-CQA, 260–480 mg kg^−1^ DW of 5-CQA, and 600–930 mg kg^−1^ DW of 4-CQA) [39]. It is worth noting that the concentration of chlorogenic acids in sweet potatoes is influenced by both the variety and the region of cultivation.

#### 3.4.2. Anthocyanins

Different anthocyanin compounds were quantified in the storage roots of all four purple-fleshed genotypes found in the Southern African region (Table 6). These metabolites’ characterisations and MS spectra are shown in Supplementary Figures S2, S12–S17. Three mono-acylated (cyanidin-caffeoyl-sophoroside-glucoside, peonidin feruloyl-sophoroside-glucoside, peonidin caffeoyl-sophoroside-glucoside) and three diacylated anthocyanins (cyanidin-caffeoyl-feruloyl-sophoroside-glucoside, peonidin-caffeoyl-hydroxybenzoyl-sophoriside-glucoside, peonidin caffeoyl-feruloyl-sophoroside-glucoside) were detected in purple sweet potato storage roots that are consumed in the Southern African region. The ratios of peonidin/cyanidin derivatives ranged from 2.16 to 6.72, indicating that peonidin-based anthocyanins were predominant in all the samples, with the 2019-1-1 sample showing the highest ratio (6.72), followed by Purple-purple pp (4.11); 16-283p (2.95) and 08-21p (2.16) were detected in purple sweet potato storage roots that are consumed in South Africa. The ratios of peonidin/cyanidin derivatives ranged from 2.16 to 6.72, indicating that peonidin-based anthocyanins were predominant in all the samples, with the 2019-1-1 sample showing the highest ratio (6.72), followed by Purple-purple pp (4.11), 16-283p (2.95) and 08-21p (2.16). Among the different purple genotypes, Purple-purple (very dark purple) contained the highest concentrations of cyanidin-caffeoyl-feruloyl-sophoroside-glucoside (230.90 mg/kg), peonidin feruloyl-sophoroside-glucoside (353.33 mg/kg), peonidin caffeoyl-sophoroside-glucoside (327.52 mg/kg), peonidin-caffeoyl-hydroxybenzoyl-sophoriside-glucoside (276.95 mg/kg), and peonidin caffeoyl-feruloyl-sophoroside-glucoside (185.25 mg/kg), while 16-283p contained cyanidin-caffeoyl-sophoroside-glucoside (123.12 mg/kg). Conversely, the ‘2019-1-1’ genotype did not exhibit peonidin caffeoyl-feruloyl-sophoroside- glucoside. In comparison to the other genotypes in this study, the cyanidin-caffeoyl-feruloyl-sophoriside-glucoside concentration in the ‘2019-1-1’ genotype was significantly lower than that in the other genotypes. On the other hand, both the 08-21p and 2019-1-1 genotypes showed significantly lower concentrations of cyanidin-caffeoyl-sophoroside-glucoside, peonidin feruloyl-sophoroside-glucoside, and peonidin caffeoyl-sophoroside-glucoside. According to Terahara et al. [40], six diacylated anthocyanins were detected in the storage roots of *Ipomoea batatas* cv. Yamagawamurasaki. The purple sweet potatoes P40 (USA genotype) contained 12 acylated anthocyanins [41]. Studies have shown that anthocyanin content varies within the different genotypes [41]. Based on the shade of colour and peonidin/cyanidin (pn/cy) ratio, sweet potato’s anthocyanin composition can be divided into two categories: cyanidins with blue domains (pn/cy >1.0) and peonidins with red domains (pn/cy > 1.0) [42]. Studies have shown that cyanidin anthocyanins have stronger antimutagenic, antioxidant, and antidepressant properties compared to peonidin anthocyanins [43,44]. The Purple-purple genotype has 20% cyanidin derivatives compared to its 80% peonidin derivatives. In contrast, 16-283p and Purple-purple have similar levels of cyanidin derivatives at 37%, while 08-21p has 22% and 2019-1-1 has 4%. Therefore, the genotype Purple-purple, with high cyanidin derivatives, would serve as a better food for physiological functions. It has been estimated that the daily intake of total anthocyanins should be between 3 and 215 mg [45]. After consumption, in 1.5 h, the plasma levels of anthocyanins were found to be from 0.5 to 1.0 µM [46]. According to Yi et al. [47], more hydroxyl groups and fewer OCH_3_ groups can decrease anthocyanin bioavailability. Until now, no study has reported the anthocyanin composition of the ‘2019-1-1 and Purple-purple genotypes. Sweet potatoes are ideal for commercial anthocyanin production because of their low cost, rapid growth cycle, and adaptability [48]. Moreover, due to their higher concentration of acyl groups, Purple-purple genotypes can withstand high temperatures and are UV stable, which makes them ideal for use as food additives.

### 3.5. The Metabolomic and Chemometric Profiles of Five Purple Sweet Potatoes Available in the Sub-Saharan African Region

**Figure 2:**
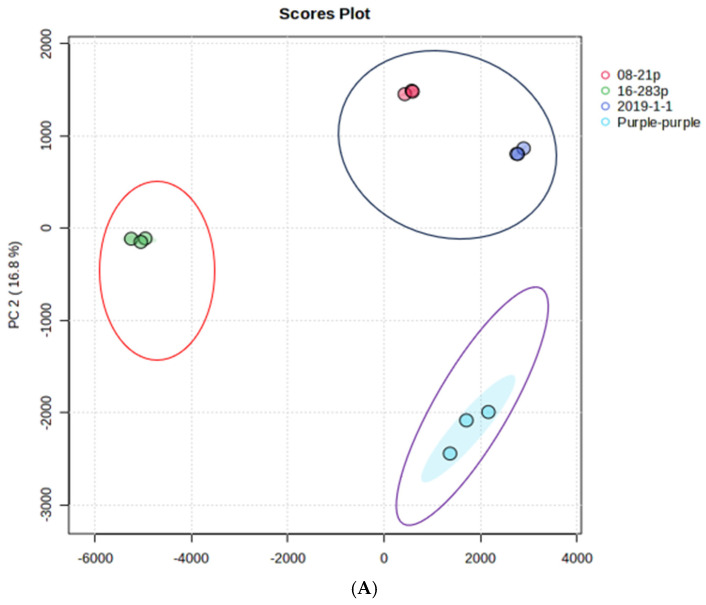

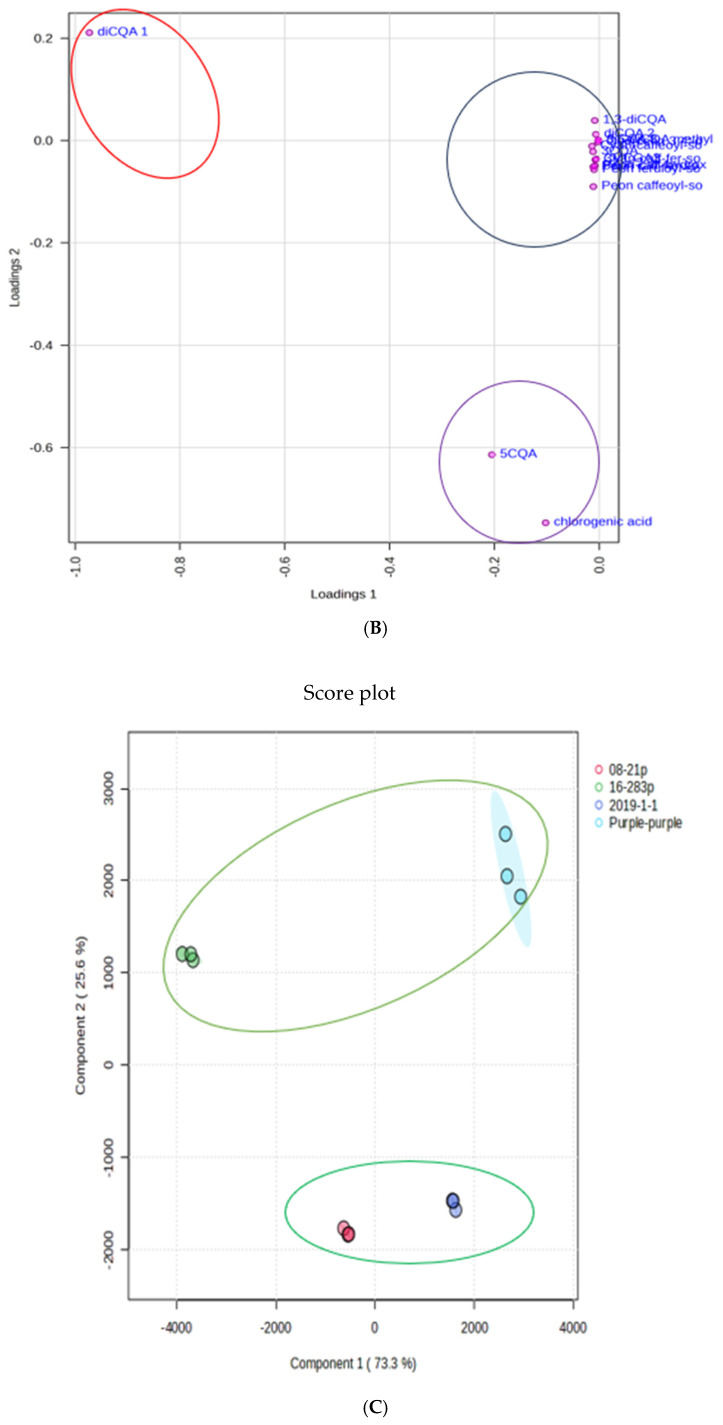

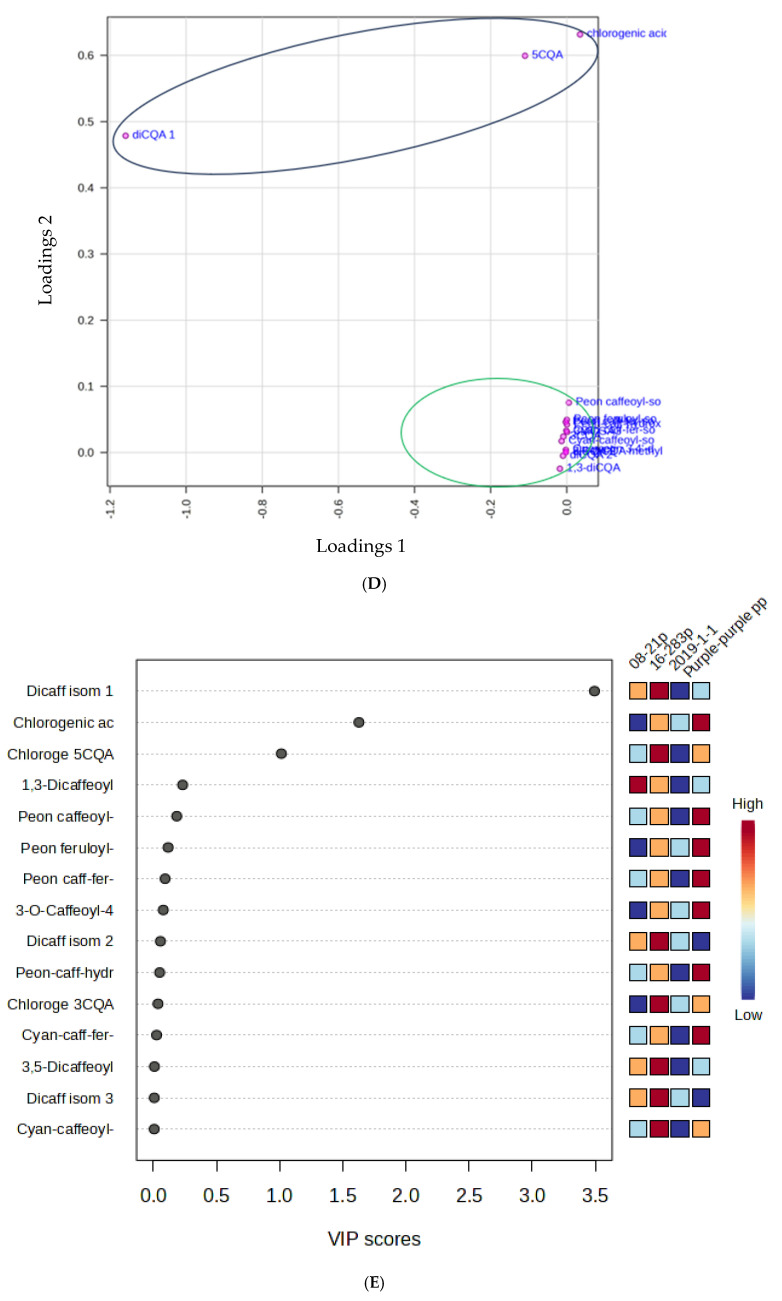

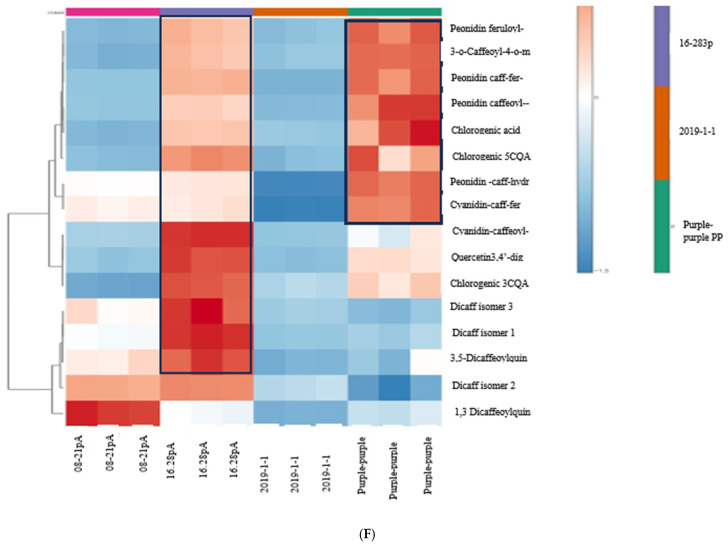
(**A**) An unsupervised PCA score plot of phenolic metabolites generated by UPLC-QTOF/MS analysis showing the separation of three clusters. (**B**) Loading of phenolic metabolites in the PCA score plot. (**C**) A PLS-DA score plot showing four sweet potato cultivars clustered into three groups. (**D**) PLS-DA score plots loaded with different phenolic compounds detected by UPLC-QTOF-MS showing two clustered groups, (**E**). Metabolites are assigned VIP scores in PLS-DA. Variable importance is determined by the score they receive from low to high. Each metabolite’s relative concentration is shown in the coloured boxes on the right. Low blue levels indicate low levels, while high red levels indicate high levels. (**F**) Heat map. In the map, the various phenolic compounds found in different sweet potato cultivars are coloured according to their concentration. The rows represent phenolic compounds, and the columns represent the sweet potato genotype. The colours red and blue indicate high and low levels, respectively.

Using UPLC-Q-TOF/MS results, unsupervised PCA analysis showed which sweet potato genotypes have the most and least phenolic compounds. Two-dimensional scatter plots of PC1 versus PC2 explained 98.9% of the variance (82.1% and 16.8%, respectively) (Figure 2A). The metabolites of the four genotypes helped to separate the four genotypes’ purple sweet potatoes into three distinct clusters, with their corresponding loadings provided in Supplementary Table S2. The loading plot (Figure 2B) revealed that the larger the distance between its point and its original point, the more a compound contributes to the total variation. Thus, the compound dicaffeoylquinic acid isomer 1 (diCQA 1), which was loaded negatively (r= −0.97) on PC1 and was the most distant from the original point, helped to separate the 16-283p genotype from the rest. Chlorogenic acid (components coefficient r = −0.75) and 5CQA (r = −0.61) were loaded negatively on PC2 and separated the genotypes Purple-purple and 16-283 from the other two genotypes. PLS-DA was utilised in this study to classify different genotypes based on their 16 metabolites. In total, 98.9% of the variation in bioactive compounds can be explained by the first two principal components (PC1 73.3% and PC2 25.6%) (Figure 2A). Three major clusters were identified based on the PLS-DA plot. Figure 2B shows the loading of different phenolic metabolites on PC1 and PC2, and the loading of the compounds is given in Supplementary Table S3. The compound diCQA 1 was loaded positively (r = 0.5) on PC2 and was distant from the original point, while 5CQA (r= 6.0) and chlorogenic acid (r= 0.63) were also loaded positively on PC2 and were able to separate the Purple-purple and 16-283 genotypes from the others. In addition to generating more accurate predictions, PLS-DA produces more meaningful models [49]. The PLS-DA model showed a high prediction level (Q2 = 0.97) as well as a high goodness-of-fit level (R^2^ = 0.82).

A variable importance in projection (VIP) score was used to evaluate the contribution of each metabolite to the separation of groups (Figure 2E). VIP scores are determined by summing the weighted PLS regression coefficients and the squares of the PLS loadings. Only the top metabolites with the highest VIP scores are considered for interpreting the results [50]. Among the top six metabolites with VIP scores >1 are chlorogenic acid 5CQA, chlorogenic acid 3CQA, and dicaffeoylquinic acid isomer 1diCQA. Diaffeoylquinic acid isomer 1 diCQA allowed us to distinguish the two groups.

In addition to the analysis, a heat map was created based on metabolite concentrations in all samples. Each row of phenolic compound data was represented by a colour block, with red boxes representing higher levels and blue boxes representing lower levels. Figure 2F displays the 16 identified metabolites from the two groups. In addition, the heat map represents the composition of phenolic metabolites in the storage roots of the four genotypes of purple sweet potato. According to the heat map, the concentration of peonidin feruloyl-sophoroside- glucoside, 3-O-caffeoyl-4-O-methylquinic acid (MCGA3), peonidin caffeoyl-sophoroside-glucoside, peonidin-caffeoyl-feruloyl-sophoriside-glucoside, chlorogenic acid 3CQA, chlorogenic acid, 5 CQA, peonidin-caffeoyl-hydroxybenzoyl-sophoriside-glucoside, and cyanidin-caffeoyl-feruloyl-sophoroside-glucoside were remarkably higher in purple genotypes. In contrast, cyanidin-caffeoyl-sophoroside-glucoside, quercetin 3,4’-diglucoside, chlorogenic acid (3CQA), dicaffeoylquinic acid isomer 1 (diCQA), dicaffeoylquinic acid isomer 3 (diCQA), 3,5-dicaffeoylquinic methyl ester, and (-)-3,5-dicaffeoylquinic methyl ester ((3,5-diCQA)) were found at higher concentrations in genotype 16-283P. Conversely, the ‘2019-1-1′ genotype showed a lower concentration of all phenolic metabolites.

### 3.6. Antioxidant Activities

Based on a genotype comparison, the Purple-purple and ‘16-283P’ genotypes had the highest antioxidant activities (FRAP, ABTS, and DPPH) (Table 7). In general, sweet potato roots with a purple colour had a higher antioxidant activity, which confirmed Ji et al.’s [51] findings.

The discrepancy in antioxidant activities and total phenolic content may be explained by the amount of rainfall that each harvest season received. In the presence of water stress, secondary metabolites can be produced to protect against oxidative stress [52]. As a result of accumulating polyphenols and acylated anthocyanins with high antioxidant activity from the very beginning of their growth, sweet potato roots are protected from biotic and abiotic stress [53].

Data present mean and standard deviation (n = 3) and Tukey’s HSD. Significant variances are indicated by distinct letters within the same column at (*p* < 0.001).

**Figure 3:**
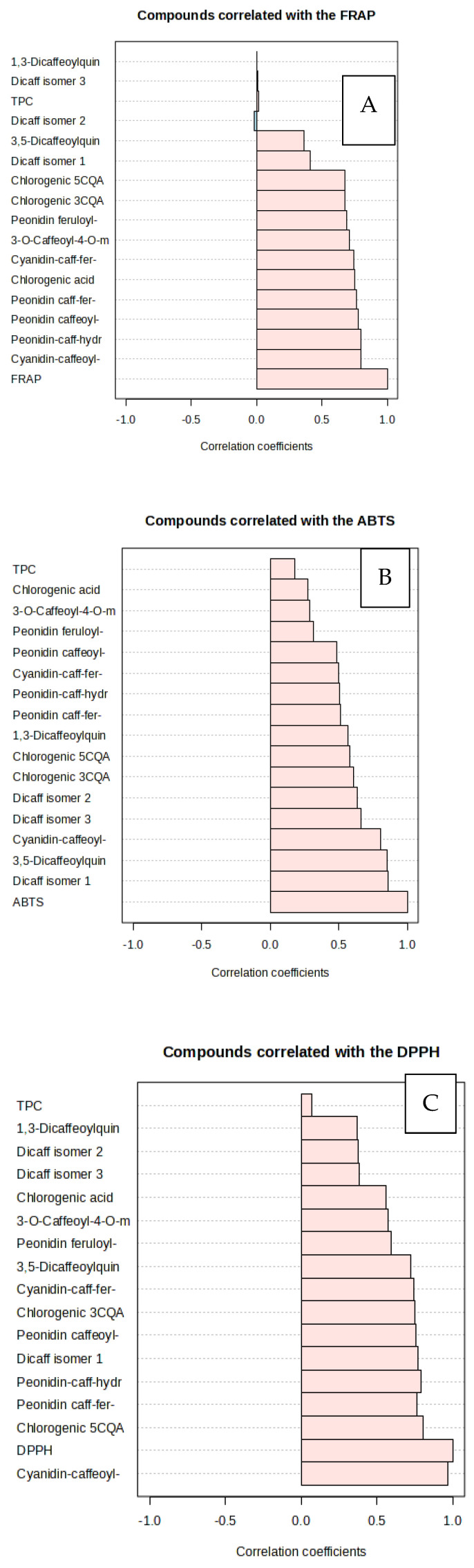
Correlation between FRAP, ABTS, DPPH and individual bioactive compounds in five genotypes of purple-fleshed sweet potato storage roots. (**A**): FRAP, (**B**): ABTS, (**C**): DPPH.

Figure 3A shows a significant correlation between individual anthocyanins and antioxidant power (FRAP). Cyanidin-caffeoyl-sophoroside-glucoside (r = 0.80, *p* < 0.05)) had the strongest correlation with the FRAP value, followed by peonidin-caffeoyl-hydroxybenzoyl-sophoriside-glucoside (r = 0.76, *p* < 0.05), peonidin caffeoyl-sophoroside- glucoside (r = 0.77, *p* < 0.05), cyanidin-caffeoyl-feruloyl-sophoroside-glucoside (r = 0.76, *p* < 0.05), chlorogenic acid (r = 0.75, *p* < 0.05), MCGA3 (r = 0.71, *p* < 0.05), peonidin feruloyl-sophoroside-glucoside (r = 0.69), 5CQA (r = 0.67), and 3CQA (r = 0.68). diCQA 1 (r = 0.86) had the highest correlation with ABTS scavenging activity (Figure 3B), followed by 3,5-dicaffeoylquinic methyl ester (r = 0.85, *p* < 0.05), cyanidin-caffeoyl-sophoroside- glucoside (r = 0.80, *p* < 0.05), diCQA 3 (r = 0.66, *p* < 0.05), diCQA 2 (r = 0.63, *p* < 0.05), 3CQA (r = 0.61, *p* < 0.05), 5CQA (r = 0.58, *p* < 0.05), 1,3-diCQA (r = 0.57, *p* < 0.05), peonidin feruloyl-sophoroside-glucoside (r = 0.51, *p* < 0.05), and peonidin-caffeoyl-hydroxybenzoyl-sophoriside-glucoside (r = 0.50, *p* < 0.05). Whereas, the DPPH scavenging activity correlated strongly (Figure 3 C) with 5CQA (r = 0.80, *p* < 0.05), peonidin feruloyl-sophoroside-glucoside (r = 0.77), peonidin-caffeoyl-hydroxybenzoyl-sophoriside-glucoside (r = 0.79, *p* < 0.05), diCQA 1 (r = 0.77, *p* < 0.05), peonidin caffeoyl-sophoroside-glucoside (r = 0.76, *p* < 0.05), 3CQA (r = 0.75, *p* < 0.05), cyanidin-caffeoyl-feruloyl-sophoroside-glucoside (r = 0.75, *p* < 0.05), 3,5-Dicaffeoylquinic methyl ester (r = 0.73, *p* < 0.05), peonidin feruloyl-sophoroside-glucoside (r = 0.59, *p* < 0.05), and MCGA3 (r = 0.57, *p* < 0.05).

Caffeoylquinic acids (chlorogenic acids, neochlorogenic acids, cryptochlorogenic acids, and isochlorogenic acids) are known to possess great antioxidant properties as free radical scavengers by donating protons or electrons by their phenolic hydroxyl groups [54]. Liao et al. [55] found a strong correlation between total anthocyanin content and antioxidant activity. Specifically, the phenolic hydroxyl group in the anthocyanin molecule plays a crucial role as a scavenger of reactive oxygen radicals. Anthocyanins have a greater antioxidant activity compared to acylated anthocyanins because the phenolic hydroxyl group in the anthocyanin structure plays a significant role in scavenging reactive oxygen radicals [56]. On the other hand, Azima et al. [57] explained that anthocyanins have been found to exhibit antioxidant activity through two primary mechanisms: the hydrogen atom donor mechanism and single electron transfer. In the hydrogen atom donor mechanism, a free radical R• removes hydrogen from the antioxidant (Ao^+^), which converts it into a more stable product. On the other hand, antioxidants (Ao^+^) donate electrons to free radicals in single electron transfer mechanisms, which reduce oxidised intermediates into stable forms. Acylated anthocyanins were more important for antioxidant activity in ABTS^+^ and FRAP activities than non-acylated forms [55].

### 3.7. Protein, Fat, Dietary Fibre

Table 8 shows significant differences in protein, fat, and dietary fibre composition between coloured sweet potato genotypes. The protein content of the sweet potato genotypes varied significantly, with the genotype Purple-purple exhibiting the highest value (28.81 ± 1.34) and the genotype 16-283P exhibiting the least value (19.12 ± 0.62) in Table 8. In addition, the total protein values (on a dry basis) were higher than those documented in the literature for sweet potatoes. Cartier et al. [58] reported a protein content of 4.07 ± 0.21–6.20 ± 0.17 g/100 g and Rodrigues et al. [59] (5.82 ± 1.43 g/100 g). Proteins play a vital role in biological activities like development and repair, depending on their bioaccessibility after digestion [58]. According to the current study, Purple-purple (28.81 g/100 g) and ‘08-21 P’ (24.31 g/100 g) are the sweet potato genotypes with the highest protein content for human nutrition in Sub-Saharan Africa. Therefore, sweet potato storage roots should be promoted and encouraged to maintain a balanced diet. A 100 g portion of Purple-purple would contribute 17.7% of a 65 kg adult male’s recommended daily protein allowance based on 0.8 g per day multiplied by weight. The protein content of the sweet potato roots of genotypes Purple-purple and ‘08-21P’ showed a higher composition than the Ethiopian genotypes G1 (Ukrewe × Ejumula-10) (7.43%), G5 (Ukrewe × Ogansagan-5) (7.29%), G6 (Resisto × Ejumula-7) (6.99%), G8 (Resisto × PIPI-2) (7.08%), and G17 (Resisto × Ogansagen-16) (6.44%), and G18 (Resisto × Ogansagen-16) (7.84%) showed the highest protein content [60].

The fat content of the storage roots was the highest in 2019-1-1 (0.83 ± 0.01 g/100 g), with genotype Purple-purple showing the lowest content of 0.47 ± 0.02 g/100 g (Table 8). On the contrary, Mitiku and Teka, [61] reported that sweet potatoes contained 1.25–1.52 g/100 g of fat and Hossain et al. [30] reported ranges of between 0.73 ± 0.05 and 2.41± 0.02 g/100 g, which were significantly higher than the results from our current study. In Brazil, a purple genotype has been reported with a lower fat content between 0.42 ± 0.04 g/100 g and 0.39 ± 0.03 g/100 g [59]. The differences can be attributed to climatic growing conditions, soil type, and location differences. Considering the recommended daily allowance for fats, a 100 g portion of genotype ‘2019-1-1′ on a fresh weight basis would contribute 1% to the daily allowance for a 65 kg adult male. Genotype ‘2019-1-1′, based on its high fat content, might be marketed as a food flavouring.

The dietary fibre content of sweet potato genotypes differed significantly, with the genotype Purple-purple having the lowest (2.51 ± 0.03 g/100 g) amount and genotype ‘08-21P’ having the highest amount (5.35 ± 0.06 g/100 g), suggesting differences in digestibility. In China [51], these values were higher, but in Brazil they were lower [32]. Soluble fibre, approximately half of the total, varied across genotypes, with ‘08-21P’ having the highest (2.48 ± 0.03 g/100 g) and ‘16-283P’ the lowest (0.45 g/100 g). The insoluble fibre was significantly higher, with ‘08-21P’ having the highest (2.87 ± 0.10 g/100 g) and ‘16-283P’ the lowest (1.95 ± 0.23 g/100 g). Soluble fibres affect digestive system transit time and glucose absorption [62], while insoluble fibres impact intestinal transit and faecal characteristics [62]. Incorporating a variety of fibre-rich foods into the diet is essential due to their distinct qualities.

### 3.8. Fe, Zn and K Content

Fe and Zn deficiencies are estimated to be 5 and 40% in Sub-Saharan Africa, respectively [63]. Sweet potato roots of the genotypes ‘2019-1-1’ and Purple-purple (Table 9) measured similar Fe levels as the Ethiopian genotypes G8 (2.55 mg per 100 g), G17 (2.16 mg per 100 g), and G19 (2.20 mg per 100 g). Iron (Fe) content ranged from 1.85 to 2.77 mg/100 g in purple sweet potato genotypes, higher than those in Benin (0.53 to 0.73 mg/100 g) [64] and orange-and-cream-flesh genotypes from South Africa (0.73 to 1.26 mg/100 g) [12]. The significant differences in Fe content may be attributed to genotype variation, as all samples were collected from the same experimental farm. Fe deficiency is linked to common micronutrient deficits such as anaemia, particularly in children, as well as vitamin A insufficiency. A 13.65 g portion of 2019-1-1 on a fresh weight basis would meet the Recommended Dietary Allowance (RDA) for iron for men, while 25.59 g for children and 30.72 g for women would meet their respective RDAs. The zinc (Zn) content ranged between 0.95 and 1.42 mg/100 g, with genotype ‘2019-1-1′ (1.42 ± 0.20 mg/100 g), ‘16-283P’ (1.32 ± 0.10 mg/100 g), and Purple-purple (1.3 ± 0.13 mg/100 g) (Table 9) exhibiting the highest composition. Nevertheless, Sanoussi et al. [64] reported a lower Zn content (0.27 mg/100 g). The trace element Zn is essential for protein synthesis, immunity, and gene expression [65]. The sweet potato root of genotype 2019-1-1 will meet the daily recommended allowance of Zn for children at 31.43 g and 22.86 g for adults. Potassium (K) content ranged from 13.3 to 22.2 mg/100 g, with cultivar 2019-1-1 having the highest K content at 22.20 mg/100 g. Hossain et al. [30] reported a range between 8.8 and 12.4 mg/100 g, which is lower than the genotypes studied here. Sanoussi et al. [64] and Senthilkumar et al. [66] reported higher levels (308.67 to 328.67 mg/100 g). K plays a role in controlling water balance, neurotransmission, and heart rate [64]. However, the 100 g sweet potato 2019-1-1 studied would contribute only 0.21% of the recommended daily allowance for adults and 0.24% of the recommended daily allowance for children.

## 4. Conclusions

Identifying the genotypes of sweet potatoes with the highest concentration of nutritional and bioactive components, the current study determined the nutrition and bioactive components of purple sweet potato roots in the Southern African region. Additionally, purple sweet potato genotypes could provide genetic resources for producing biofortified purple-fleshed sweet potato genotypes to compensate for macronutrient Fe and Zn deficiencies. The study found that the phenolic compounds in the roots of four sweet potato genotypes were almost indistinguishable, but their compositions varied. A study identified the local purple sweet potato genotype Purple-purple as a potential source of antioxidants and protein, and ‘2019-1-1’ for the dietary minerals Fe and Zn. Furthermore, it would be ideal to recommend purple sweet potato genotypes available in the Southern African region for consumption. Moreover, these genotypes must be tested in different environments to ensure stable yields.

## Figures and Tables

**Table 1:** Morphological characteristics of four genotypes of purple-fleshed sweet potato storage roots.

Purple Sweet Potato Genotypes	Skin Colour	Flesh Colour	Shape	Surface Defects
‘08-21P’	Light purple	Violet, pink-cream	Long elliptic	Absent
‘2019-1-1′	Purple	Purple, cream ring	Obovate–elliptic	Grooves, some cracks
Purple-purple	Dark purple	Very dark purple	Long elliptic	Long thick veins
‘16-283P’	Light purple	Dark purple, slight ring	Long elliptic	Absent

**Table 2:** Yield, length, diameter, and cortex of the four different genotypes of purple sweet potato storage roots.

Genotype	Yield (kg/20 Plants) S1	Length (cm)	Diameter (cm)	Cortex (mm)
‘08-21P’	20.64+ 1.90 ^a^	21.80 ± 2.88 ^a^	5.07 ± 1.28 ^a^	3.40 ± 1.17 ^ab^
‘2019-1-1′	5.45 ± 1.26 ^d^	16.85 ± 3.35 ^bc^	5.48 ± 1.17 ^a^	1.16 ± 0.31 ^c^
Purple-purple	15.48 ± 2.43 ^b^	20.40 ± 3.85 ^ab^	4.26 ± 0.83 ^b^	3.85 ± 1.03 ^a^
‘16-283P’	7.56 ± 1.72 ^c^	17.00 ± 3.63 ^bc^	4.18 ± 0.91 ^b^	2.80 ± 0.75 ^b^
LSD	2.81	7.19	2.19	1.65

Data present mean and standard deviation (n = 10) and Tukey’s HSD of four purple-fleshed sweet potato storage root genotypes. Significant variances are indicated by distinct letters within the same column at (*p* < 0.001).

**Table 3:** Flesh colour values of four genotypes of purple-fleshed sweet potato storage roots.

Genotypes	L*	a*	b*
‘08-21P’	45.58 ± 3.50 ^a^	30.58 ± 5.14 ^a^	5.57 ± 2.20 ^a^
‘2019-1-1′	42.69 ± 5.94 ^b^	8.64 ± 3.02 ^c^	5.18 ± 4.98 ^b^
Purple-purple	28.82 ± 2.74 ^d^	18.36 ± 3.18 ^b^	3.09 ± 0.51 ^c^
‘16-283P’	34.79 ± 3.39 ^c^	8.12 ± 1.11 ^c^	2.21 ± 2.64 ^cd^
LSD	4.4	3.79	3.3

Data present mean and standard deviation (n = 3) and Tukey’s HSD. Significant variances are indicated by distinct letters within the same column at (*p* < 0.001).

**Table 4:** Total phenolic content of four genotypes of purple-fleshed sweet potato storage roots.

Genotypes	Total Phenolic (CAE mg/g) DW
‘08-21P’	43.54 ± 7.90 ^b^
‘2019-1-1′	50.69 ± 1.48 ^a^
Purple-purple	42.45 ± 5.31 ^b^
‘16-283P’	53.24 ± 3.86 ^a^
LSD	8.72

Data present mean and standard deviation (n = 3) and Tukey’s HSD. Significant variances are indicated by distinct letters within the same column at (*p* < 0.001). (CAE mg/g): chlorogenic acid equivalent; DW: dry weight.

**Table 5:** Identification and quantification of cinnamic acids and derivatives and flavonoid composition of purple-coloured sweet potato (*Ipomoea batatas* L.) genotypes found in Sub-Saharan Africa by UPLC–QTOF/MS.

RT	[M-H]^−^ (*m*/*z*)	MSE Fragments	Molecular Formula	Tentative Identification	Sweet Potato Genotypes (Roots)			
					‘16-283P’	‘08-21P’	‘2019-1-1′	Purple-purple
					Concentrations of cinnamic acids and derivatives in mg/kg			
4.44	353.087	135.043 179.034 191.055 201.016	C_16_H_18_O_9_	Chlorogenic acid 3CQA 3-*O*-Caffeoylquinic acid	246.26 ^a^ ± 3.54	118.81 ^d^ ± 1.81	147.41 ^c^ ± 3.22	198.26 ^b^ ± 1.19
5.29	707.182	135.043 161.022 179.034 191.055 353.088 707.183	C_16_H_18_O_9_	Chlorogenic acid 3CQA 3-*O*-Caffeoylquinic acid	2201.27 ^b^ ± 2.02	450.30 ^c^ ± 2.25	670.03 ^c^ ± 2.37	3032.33 ^a^ ± 1.46
5.29	353.087	135.043 161.023 179.034 191.055 353.088	C_16_H_18_O_9_	Chlorogenic acid 5CQA 5-*O*-Caffeoylquinic acid (Neochlorogenic acid)	4354.93 ^a^ ± 1.66	2195.08 ^b^ ± 2.43	2178.12 ^b^ ± 2.60	4210.58 ^a^ ± 2.52
6.48	367.103	191.056	C_17_H_20_O_9_	3-O-Caffeoyl-4-O-methylquinic acid (MCGA3)	184.71 ^b^ ± 5.14	95.49 ^d^ ± 2.89	106.71 ^c^ ± 2.53	221.10 ^a^ ± 1.73
7.41	515.119	135.043 161.023 173.044 179.033 191.055 201.016 335.076 353.087 388.996	C_25_H_24_O_12_	1,3-Dicaffeoylquinic acid (1,3-diCQA)	222.45 ^b^ ± 1.03	433.27 ^a^ ± 1.28	98.34 ^d^ ± 2.45	179.91 ^c^ ± 1.34
7.60	515.119	135.043 179.033 191.055 353.087 375.069	C_25_H_24_O_12_	Dicaffeoylquinic acid isomer 1 diCQA	10547.26 ^a^ ± 1.44	5420.71 ^b^ ± 1.98	3068.42 ^c^ ± 2.02	3467.92 ^c^ ± 3.07
8.04	515.119	173.044 191.054 353.088 375.070	C_25_H_24_O_12_	Dicaffeoylquinic acid isomer 2 diCQA	124.65 ^a^ ± 0.23	118.34 ^a^ ± 1.11	82.08 ^b^ ± 1.84	61.58 ^c^ ± 1.90
8.68	515.119	179.034 191.055 339.050 353.087 375.070	C_25_H_24_O_12_	Dicaffeoylquinic acid isomer 3 diCQA	18.08 ^a^ ± 1.10	11.48 ^b^ ± 0.88	7.19 ^c^ ± 0.20	6.33 ^c^ ± 0.49
8.89	529.136	367.104 375.167 397.146 519.331	C_26_H_26_O_12_	3,5-Dicaffeoylquinic methyl ester; (-)-3,5-Dicaffeoylquinic methyl ester 3,5-diCQA)	22.13 ^a^ ± 1.01	14.76 ^b^ ± 0.86	6.66 ^c^ ± 0.32	9.43 ^c^ ± 3.44
					Concentrations of flavonoids in mg/kg			
6.28	625.141	300.027 301.032 339.051 371.098 471.152 533.129 555.112	C_27_H_30_O_17_	Quercetin 3,4′-diglucoside	29.23 ^a^ ± 0.71	8.54 ^c^ ± 0.52	7.86 ^c^ ± 0.23	18.88 ^b^ ± 0.46

Means followed by the same letter within the row are not significantly different (*p* < 0.05); each of the samples was replicated three times, and the results are expressed as mean ± standard deviation.

**Table 6:** Identification and quantification of anthocyanin composition of purple-coloured sweet potato (*Ipomoea batatas* L.) genotypes found in Sub-Saharan Africa by UPLC–QTOF/MS.

RT	[M-H]^−^ (*m*/*z*)	MSE Fragments	M-H Formula	Identified Compound	Sweet Potato Genotypes (Roots)			
					‘16-283P’	‘08-21P’	‘2019-1-1′	Purple-purple
					Concentrations in mg/kg			
5.66	933.231	287.055	C_42_H_45_O_24_	Cyanidin-caffeoyl-sophoroside-glucoside	123.12 ^a^ ± 1.13	18.17 ^c^ ± 0.30	11.54 ^c^ ± 0.75	47.25 ^b^ ± 1.48
7.00	1109.278	287.055	C_52_H_53_O_27_	Cyanidin-caffeoyl-feruloyl-sophoroside-glucoside	159.22 ^b^ ± 2.79	151.45 ^b^ ± 3.48	17.91 ^c^ ± 0.52	230.90 ^a^ ± 1.10
6.47	961.263	301.071	C_44_H_49_O_24_	Peonidin feruloyl-sophoroside-glucoside	301.27 ^b^ ± 1.5	160.03 ^c^ ±1.79	167.06 ^c^ ± 5.11	353.33 ^a^ ± 1.97
6.50	947.247	301.091	C_43_H_47_O_24_	Peonidin caffeoyl-sophoroside-glucoside	203.34 ^b^ ± 1.46	28.42 ^c^ ± 1.73	12.71 ^c^ ± 0.95	327.52 ^a^ ± 1.69
6.90	1067.268	301.071 463.123	C_50_H_51_O_26_	Peonidin-caffeoyl-hydroxybenzoyl-sophoriside-glucoside	180.97 ^b^ ± 1.62	159.41 ^c^ ± 1.85	18.05 ^d^ ± 0.76	276.95 ^a^ ± 1.70
7.03	1123.293	301.071 463.124	C_53_H_55_O_27_	Peonidin caffeoyl-feruloyl-sophoroside-glucoside	147.9 ^b^ ± 1.75	19.13 ^c^ ± 0.35	0^c^	185.25 ^a^ ± 1.16

Means followed by the same letter within the row are not significantly different (*p* < 0.05); each of the samples was replicated three times, and the results are expressed as mean ± standard deviation.

**Table 7:** Antioxidant activities of four different genotypes of purple-fleshed sweet potato storage roots.

Genotype	FRAP TEAC mM/g	ABTS IC_50_ Value mg/g	DPPH IC_50_ Value mg/g
‘08-21P’	16.03 ± 0.83 ^b^	1.68 ± 0.07 ^b^	1.84 ± 0.07 ^c^
‘2019-1-1′	14.56 ± 2.43 ^b^	1.99 ± 0.11 ^b^	2.31 ± 0.15 ^d^
Purple-purple	21.23 ± 1.51 ^a^	1.50 ± 0.09 ^a^	1.41 ± 0.01 ^b^
‘16-283P’	21.19 ± 1.80 ^a^	1.21 ± 0.02 ^a^	1.05 ± 0.02 ^a^
LSD	2.97	0.60	0.28

Data present mean and standard deviation (n = 3) and Tukey’s HSD. Significant variances are indicated by distinct letters within the same column at (*p* < 0.001).

**Table 8:** Protein, fat, and dietary fibre composition of four different, purple-fleshed sweet potato storage root genotypes (DW).

Genotypes	Fat g/100 g	Protein g/100 g	Total Dietary Fibre g/100 g
‘08-21 P’	0.75 ± 0.01 ^b^	24.31 ± 1.25 ^b^	5.35 ± 0.06 ^a^
‘2019-1-1′	0.83 ± 0.01 ^a^	21.44 ± 1.31 ^c^	3.81 ± 0.01 ^b^
Purple-purple	0.47 ± 0.02 ^c^	28.81 ± 1.34 ^a^	2.51 ± 0.03 ^c^
‘16-283P’	0.76 ± 0.10 ^b^	19.12 ± 0.62 ^d^	2.86 ± 0.12 ^c^
LSD	0.02	0.34	0.78

Data present mean and standard deviation (n = 3) and Tukey’s HSD. Significant variances are indicated by distinct letters within a same column at (*p* < 0.001).

**Table 9:** Iron, zinc, and potassium composition of four different, purple-fleshed sweet potato storage root genotypes.

	K (mg/100 g)	Fe mg/100 g	Zn mg/100 g
‘08-21P’	17.2 ± 0.11 ^bc^	2.08 ± 0.05 ^d^	0.95 ± 0.09 ^c^
‘2019-1-1′	22.2 ± 0.06 ^a^	2.77 ± 0.06 ^a^	1.42 ± 0.20 ^a^
Purple-purple	16.9 ± 0.04 ^bc^	2.17 ± 0.08 ^c^	1.3 ± 0.13 ^a^
‘16-283P’	16.8 ± 0.11 ^c^	2.60 ± 0.13 ^b^	1.32 ± 0.10 ^a^
CV%	5.2	3.18	10.23
LSD	0.17	0.20	0.24

Data present mean and standard deviation (n = 3) and Tukey’s HSD. Significant variances are indicated by distinct letters within a same column at (*p* < 0.001). Key: Fe: Iron, K: Potassium, Zn: Zinc.

## Data Availability

Data will be made available upon request.

## Supplementary Materials

The following supporting information can be downloaded at: https://www.mdpi.com/article/10.3390/antiox13030338/s1, Figure S1: MS chromatogram of sweet potato roots with identified phenolic compounds. Figure S2: MS/MS chromatogram overlaid on the MS and UV chromatograms of sweet potato roots with identified anthocyanins. Figure S3: Illustrating the increase/decrease of phenolic acids in the different genotypes of sweet potato roots. Figure S4: MS spectrum of 3CQA overlaid on its MS/MS and UV spectra adjacent to its chemical structure. Figure S5: MS spectrum of 5CQA overlaid on its MS/MS and UV spectra adjacent to its chemical structure. Figure S6: MS spectrum of quercetin 3,4’-diglucoside overlaid on its MS/MS and UV spectra adjacent to its chemical structure. Figure S7: MS spectrum of 3-*O*-caffeoyl-4-*O*-methylquinic acid overlaid on its MS/MS and UV spectra adjacent to its chemical structure. Figure S8: Showing the MS spectrum of 1,3-diCQA overlaid on its MS/MS and UV spectra adjacent to its chemical structure. Figure S9: MS spectrum of diCQA 1 overlaid on its MS/MS and UV spectra adjacent to its chemical structure. Figure S10: Showing the MS spectrum of 4,5-diCQA overlaid on its MS/MS and UV spectra adjacent to its chemical structure. Figure S11: Showing the MS spectrum of 3,5-dicaffeoylquinic methyl ester overlaid on its MS/MS and UV spectra adjacent to its chemical structure. Figure S12: MS spectrum of cyanidin-caffeoyl-sophoroside-glucoside overlaid on its MS/MS and UV spectra adjacent to its chemical structure. Figure S13: MS spectrum of peonidin feruloyl-sophoroside-glucoside overlaid on its MS/MS spectrum adjacent to its chemical structure. Figure S14: MS spectrum of peonidin caffeoyl-sophoroside-glucoside overlaid on its MS/MS and UV spectra adjacent to its chemical structure. Figure S15: MS spectrum of cyanidin-caffeoyl-feruloyl-sophoroside-glucoside overlaid on its MS/MS and UV spectra adjacent to its chemical structure. Figure S16: MS spectrum of peonidin-caffeoyl-hydroxybenzoyl-sophoroside-glucoside overlaid on its MS/MS and UV spectra adjacent to its chemical structure. Figure S17: MS spectrum of peonidin caffeoyl-feruloyl-sophoroside-glucoside overlaid on its MS/MS and UV spectra adjacent to its chemical structure. Table S1: Regression equations, retention time, LOD, and LOQ for anthocyanins and phenolic compounds quantified using TargetLynx. Table S2: Principal component analysis (PCA) showing the loadings of phenolic metabolites on PC1 and PC2. Table S3: Partial least squares–discriminant analysis (PLS-DA) showing the loadings of phenolic metabolites on PC1 and PC2.
