# Supplementary material for: Phytonutritional Composition and Antioxidant Properties of Southern African, Purple-Fleshed Sweet Potato (Ipomoea batatas (L.) Lam.) Storage Roots
Source: Antioxidants (Basel). 2024 Mar 11;13(3):338. doi: 10.3390/antiox13030338 (PMC10968400; doi:10.3390/antiox13030338)
Supplement: Supplementary file 1 [file antioxidants-13-00338-s001.zip › antioxidants-2895109-supplementary.pdf]

Phytonutritional composition and antioxidant properties of Southern African, purple-fleshed sweet potato (*Ipomoea batatas* (L.) Lam.) storage roots

Ayanda Ngcobo, A[1]., Mianda Sephora, Faith Seke, Sunette M. Laurie, [2] & Dharini Sivakumar[1,3]

[1] Department of Crop Sciences, Tshwane University of Technology, Pretoria, 0183, South Africa

[2]Agricultural Research Council, Industrial and Medicinal Plants, 293, South Africa

[3] Centre for Nutrition and Food Sciences, Queensland Alliance for Agriculture and Food Innovation, Indooroopilly QLD 4068, Australia

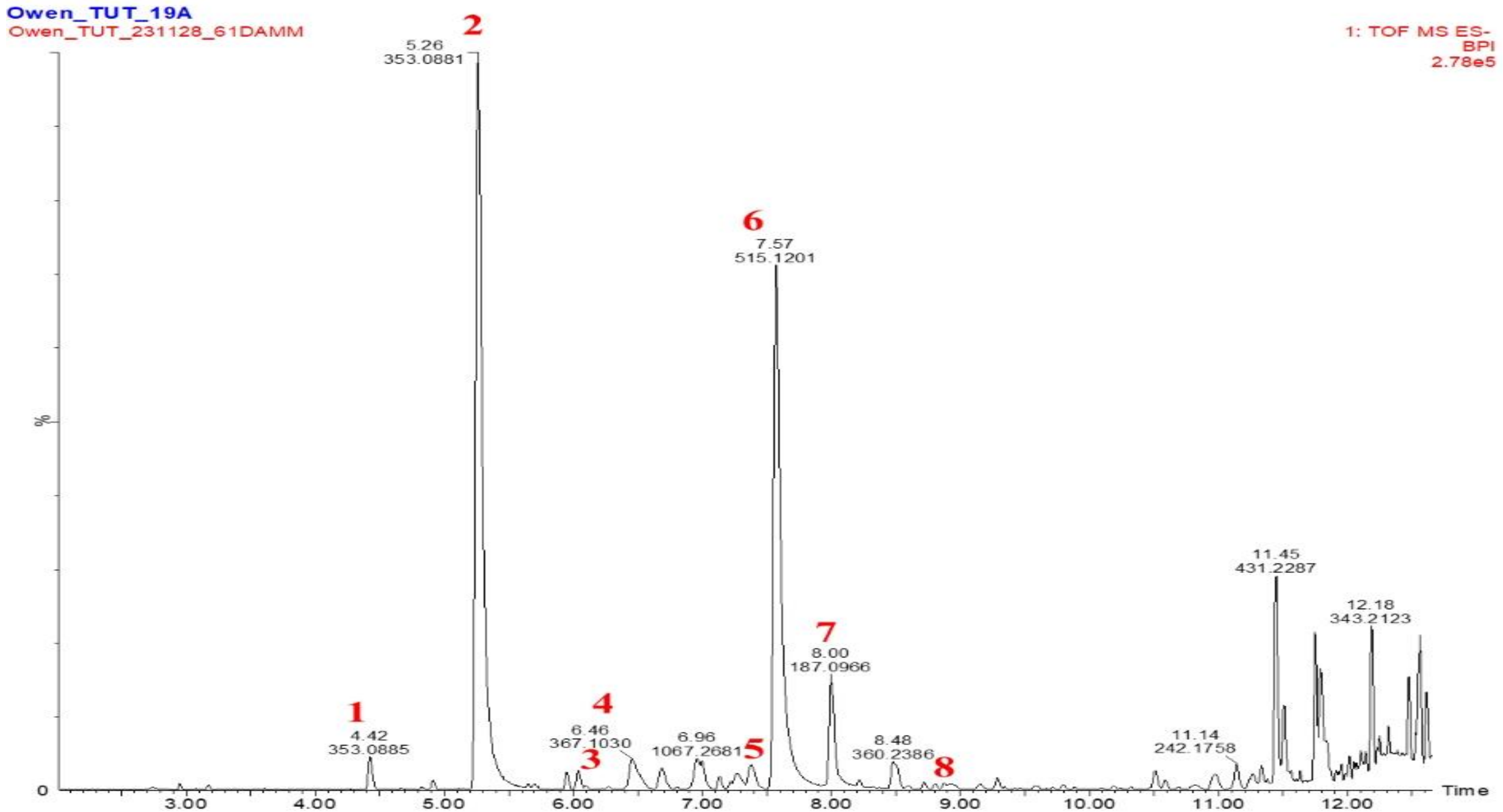

Figure S1: MS chromatogram of sweet potatoes roots with identified phenolic compounds: 3CQA (Peak 1), 5CQA (Peak 2), quercetin 3,4'-diglucoside (Peak 3), 3-O-caffeoyl-4-O-methylquinic acid (Peak 4), 1,3-diCQA (Peak 5), diCQA 1 (Peak 6), 4,5-diCQA (Peak 7), 3,5-dicaffeoylquinic methyl ester (Peak 8).

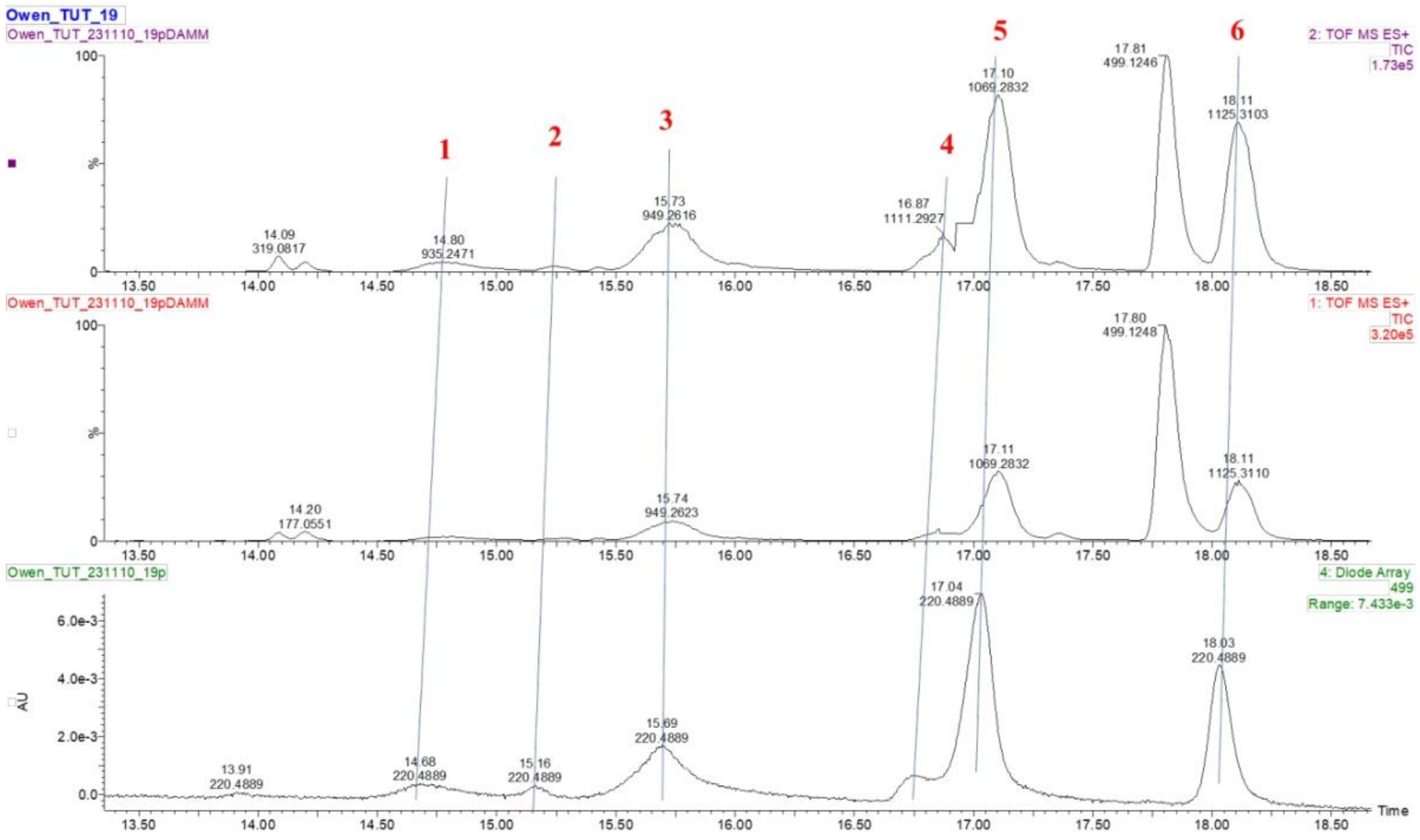

Figure S2: MS/MS chromatogram overlaid on the MS and UV chromatograms of sweet potatoes roots with identified anthocyanins: cyanidin-caffeoyl-sophoroside-glucoside (Peak 1), peonidin feruloyl-sophoroside-glucoside (Peak 2), peonidin caffeoyl-sophoroside-glucoside (Peak 3), cyanidin-caffeoyl-feruloyl-sophoroside-glucoside (Peak 4), peonidin-caffeoyl-hydroxybenzoyl-sophoriside-glucoside (Peak 5), peonidin caffeoyl-feruloyl-sophoroside-glucoside (Peak 6).

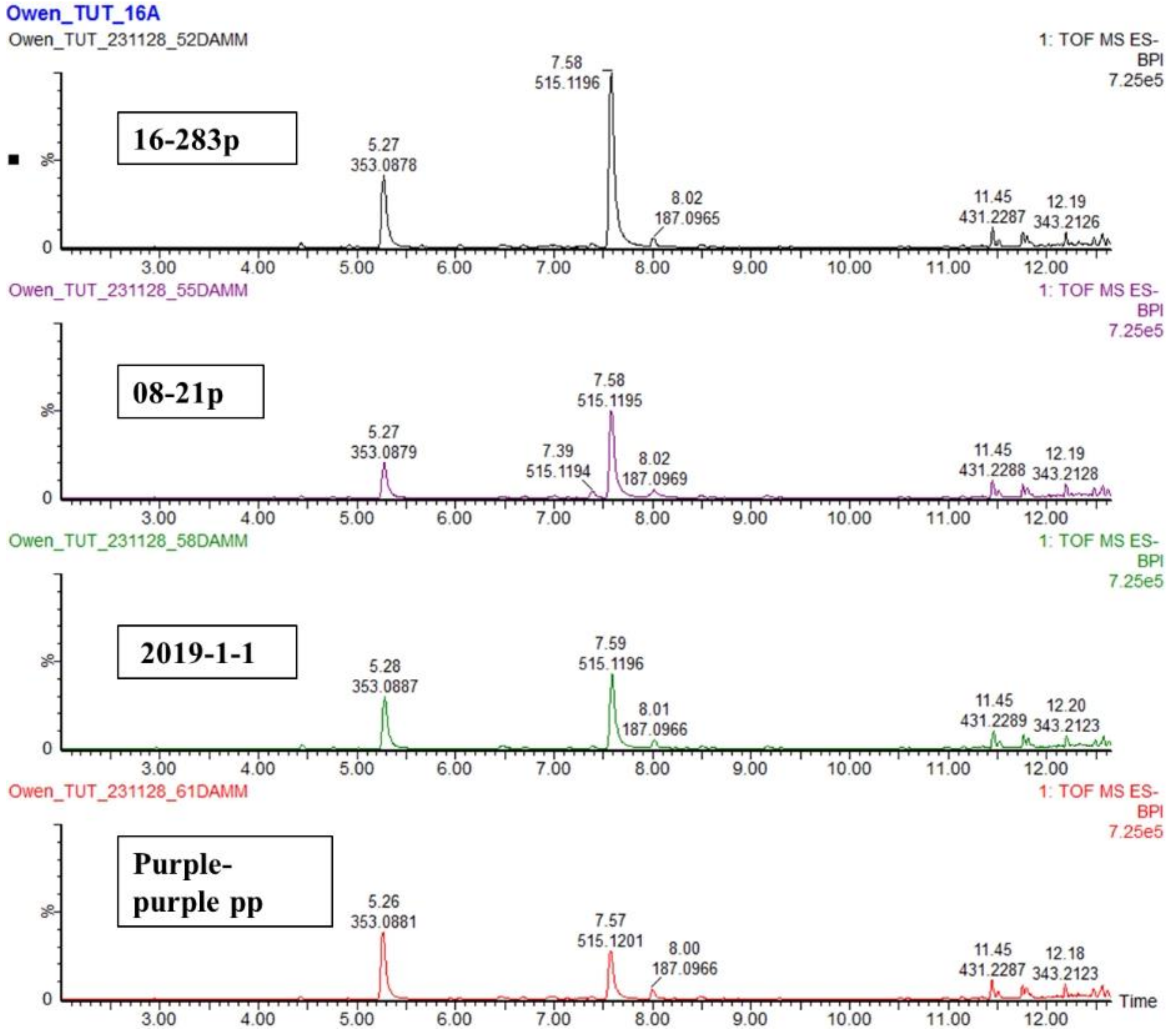

Figure S3: Illustrating the increase/decrease of phenolic acids in the different genotypes of sweet potatoes roots.

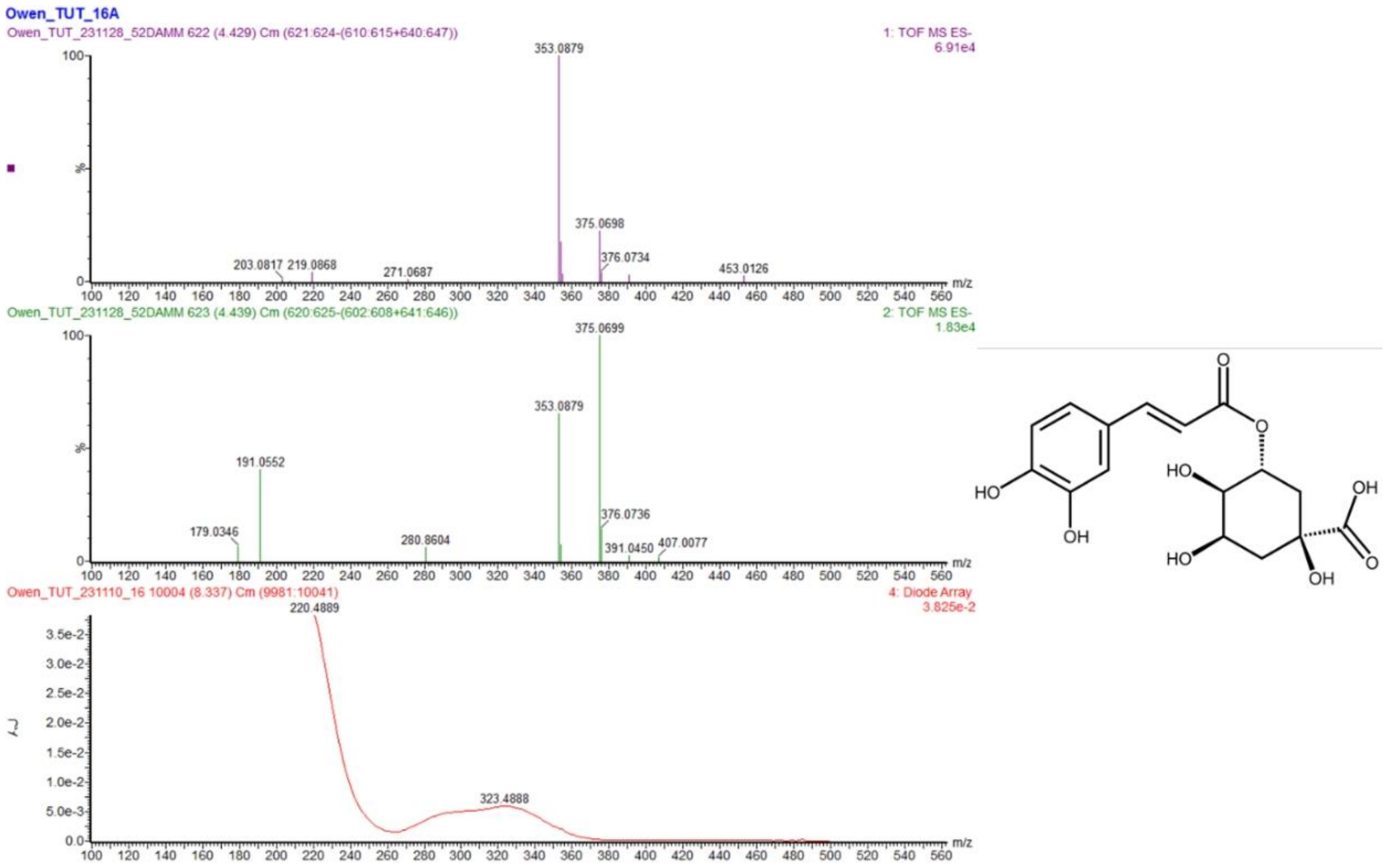

Figure S4: MS spectrum of 3CQA overlaid on its MS/MS and UV spectra adjacent to its chemical structure.

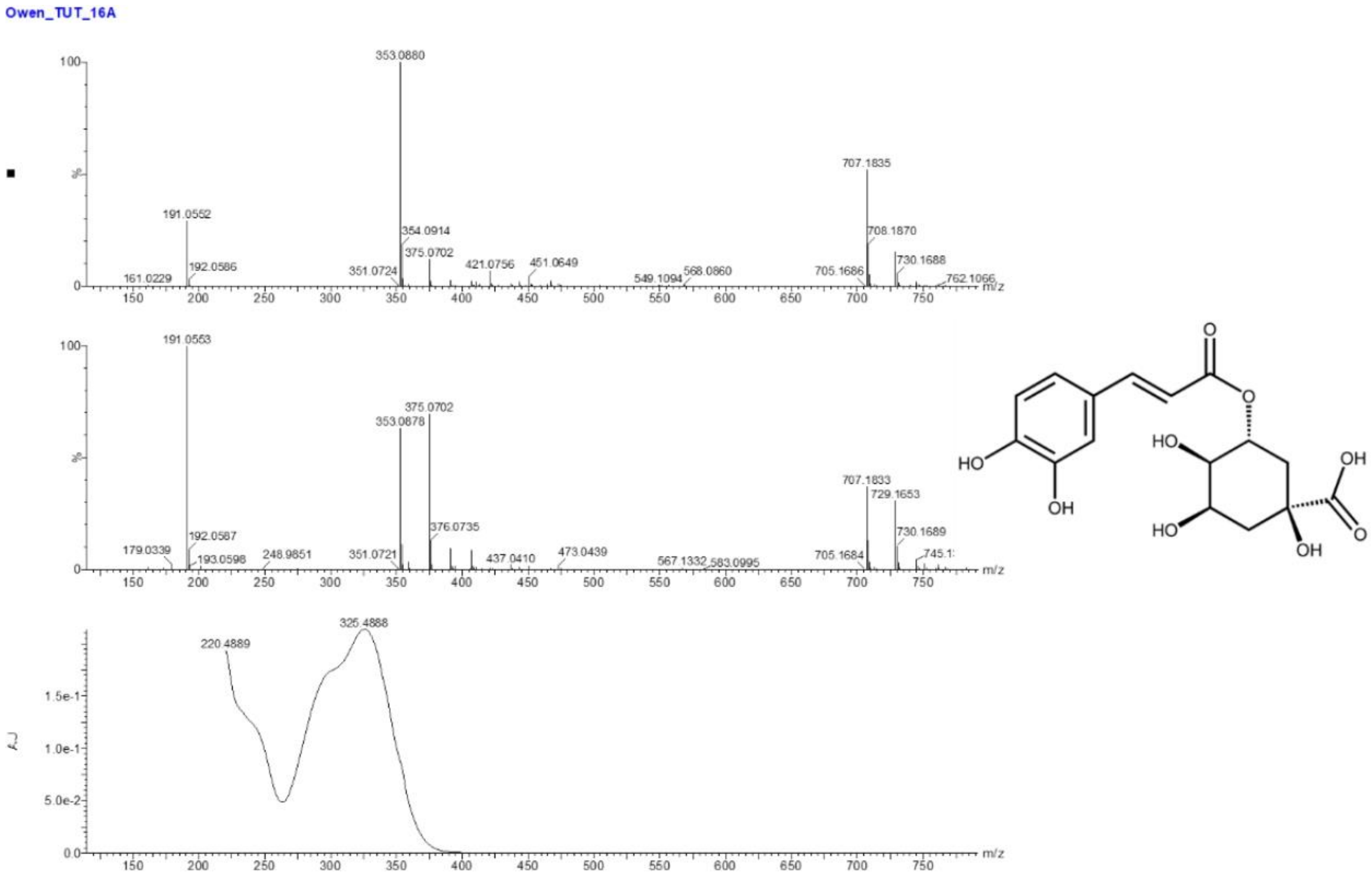

Figure S5: MS spectrum of 5CQA overlaid on its MS/MS and UV spectra adjacent to its chemical structure.

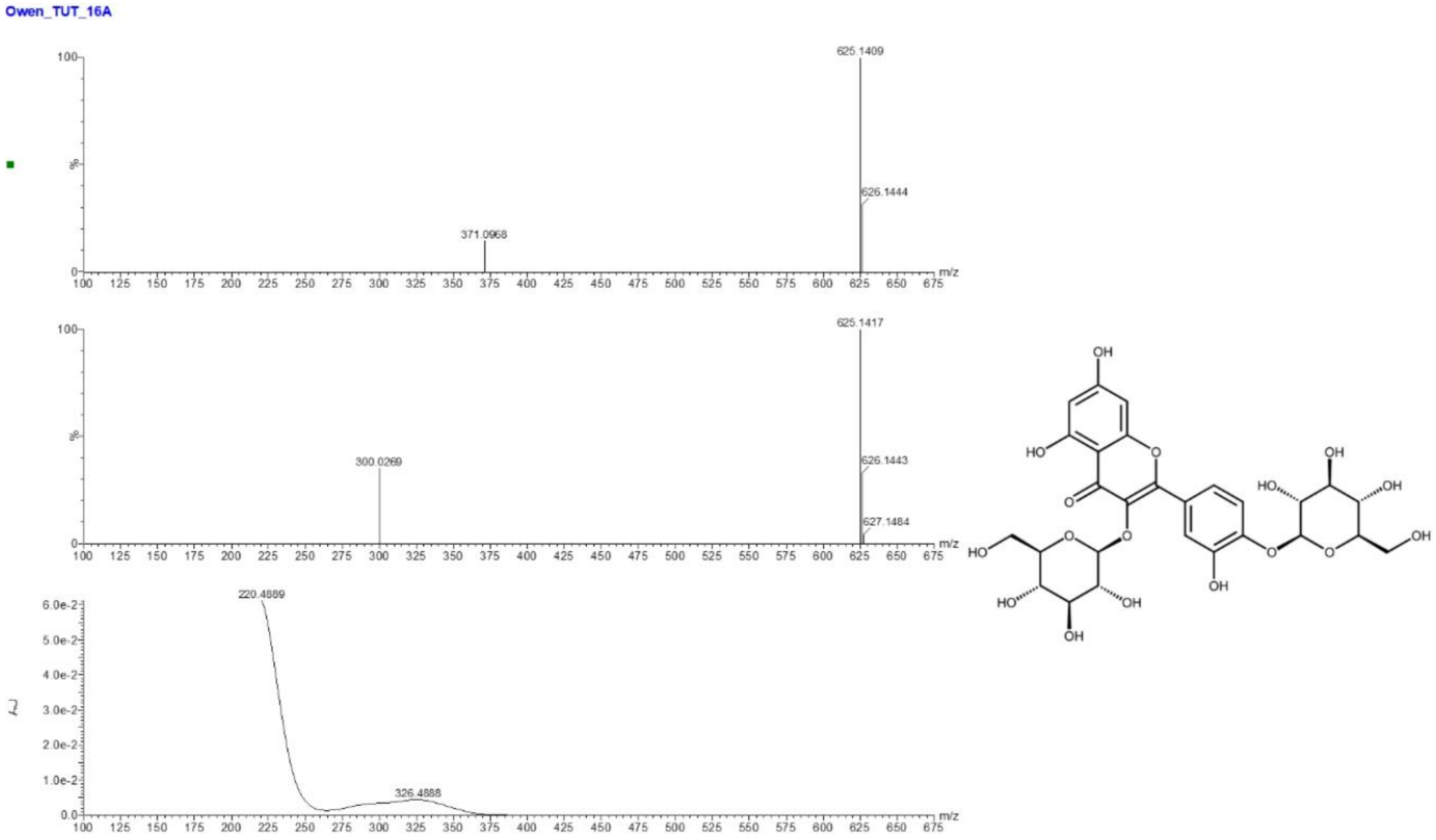

Figure S6: MS spectrum of quercetin 3,4'-diglucoside overlaid on its MS/MS and UV spectra adjacent to its chemical structure.

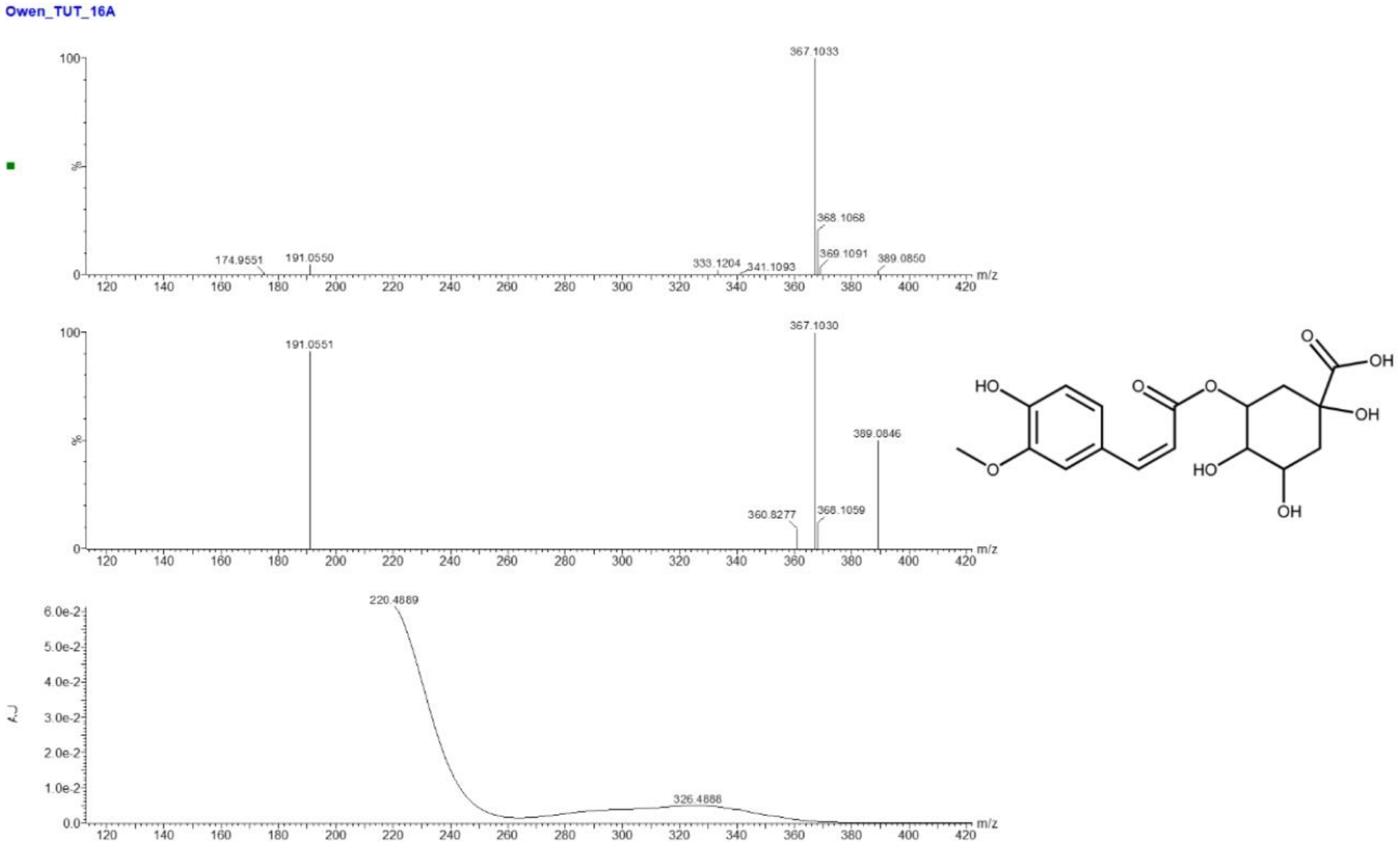

Figure S7: MS spectrum of 3-*O*-caffeoyl-4-*O*-methylquinic acid overlaid on its MS/MS and UV spectra adjacent to its chemical structure.

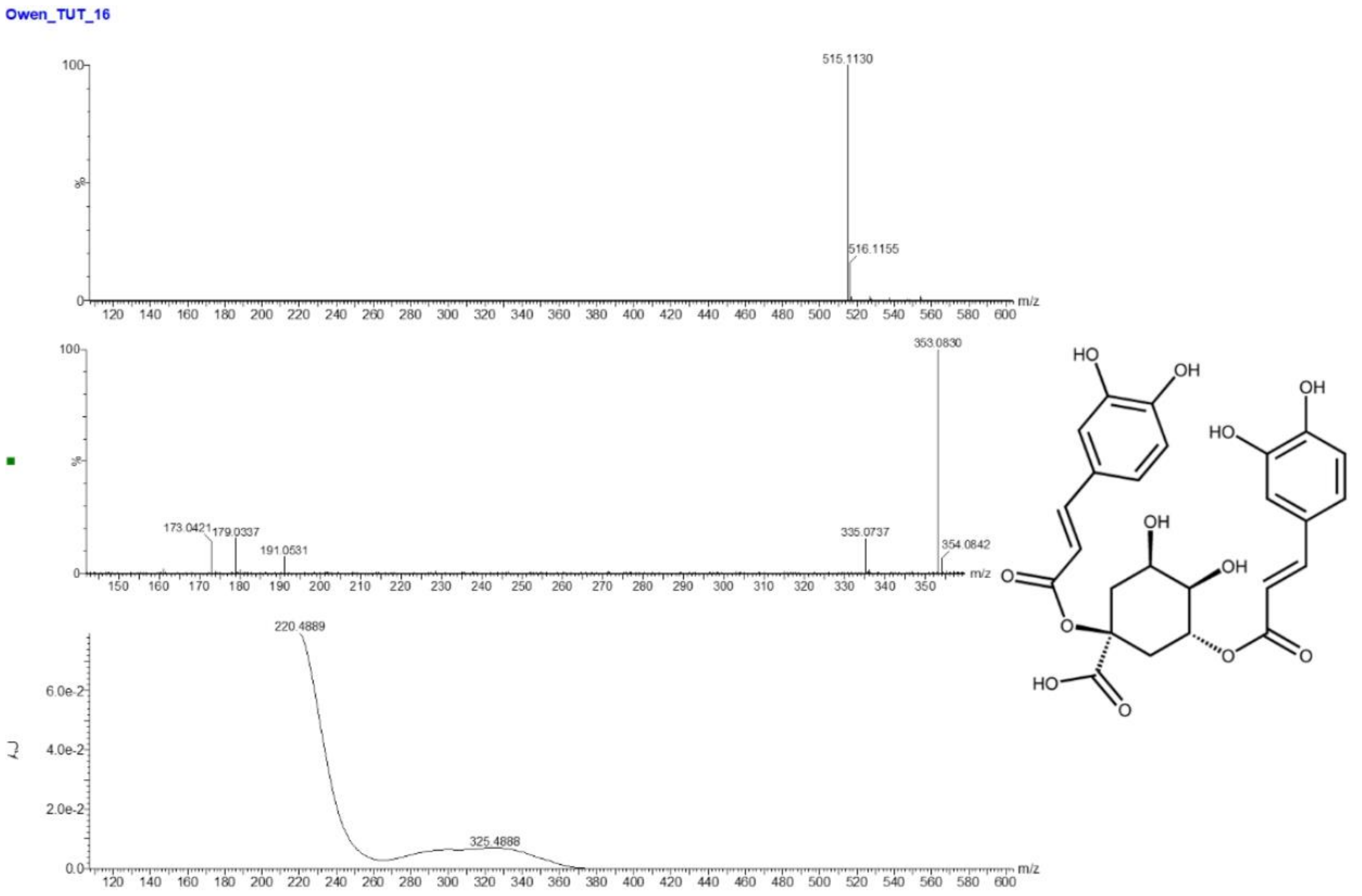

Figure S8: Showing the MS spectrum of 1,3-diCQA overlaid on its MS/MS and UV spectra adjacent to its chemical structure.

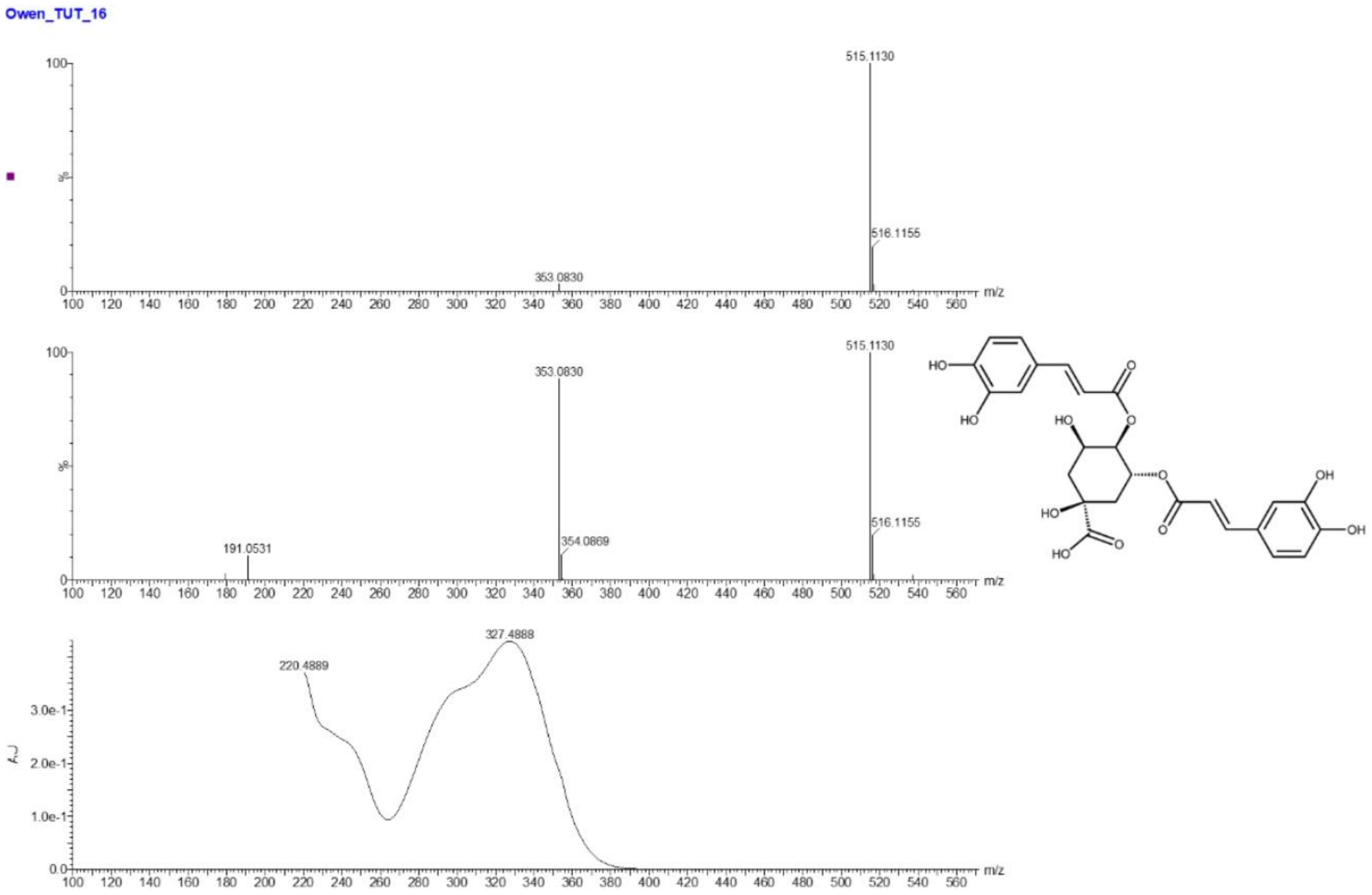

Figure S9: MS spectrum of diCQA 1 overlaid on its MS/MS and UV spectra adjacent to its chemical structure.

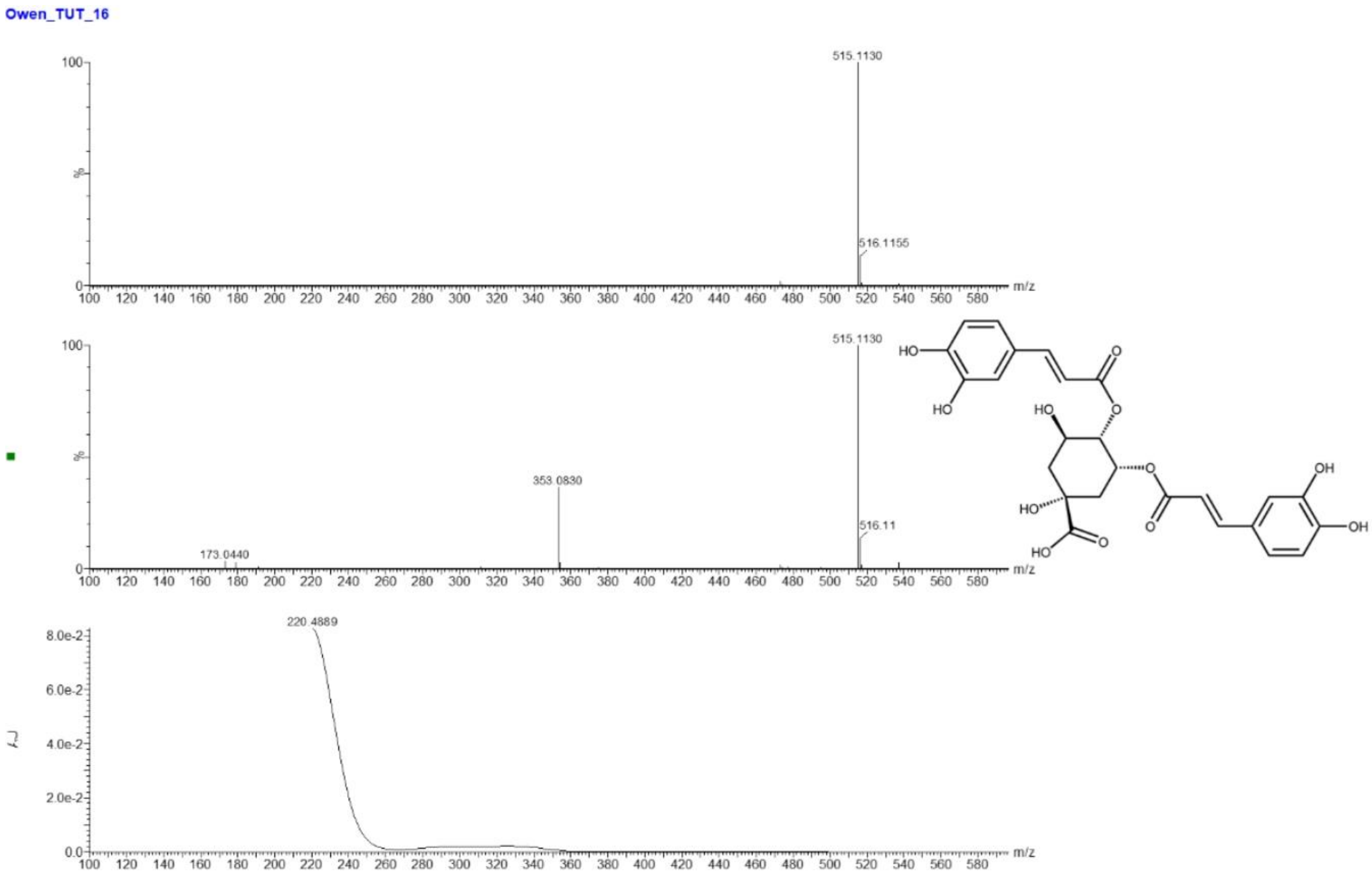

Figure S10: MS spectrum of 4,5-diCQA overlaid on its MS/MS and UV spectra adjacent to its chemical structure.

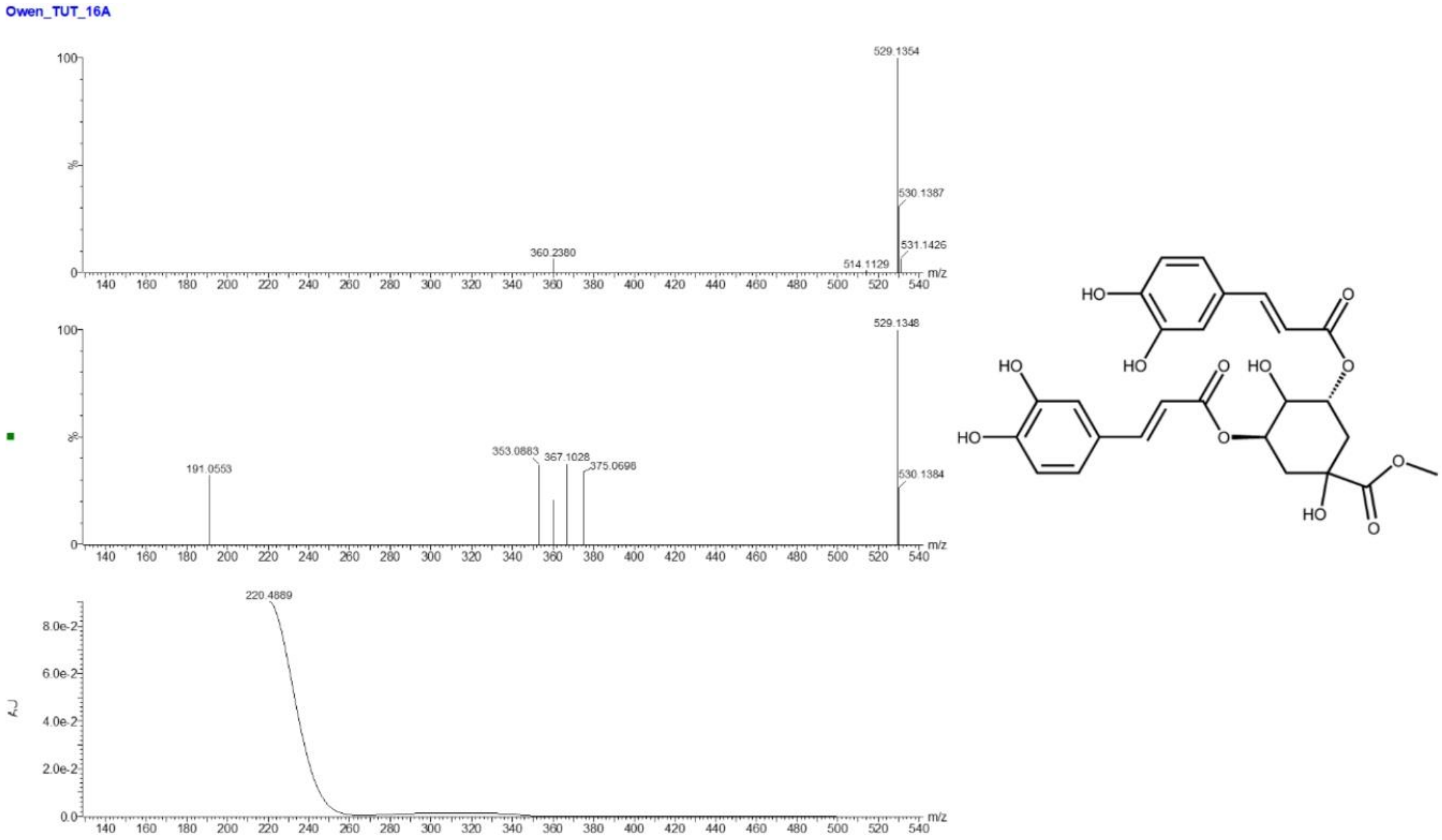

Figure S11: MS spectrum of 3,5-dicaffeoylquinic methyl ester overlaid on its MS/MS and UV spectra adjacent to its chemical structure.

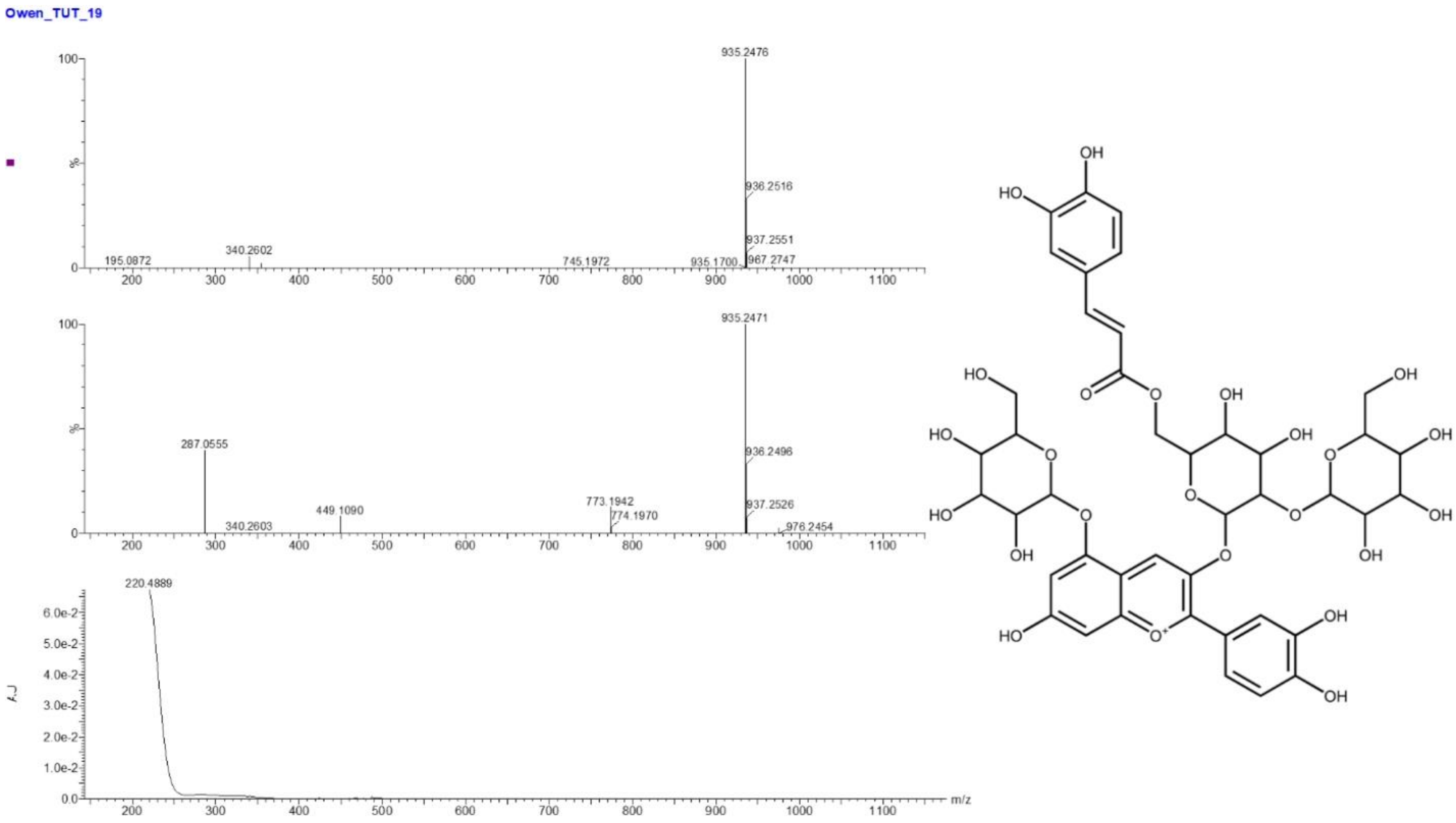

Figure S12: MS spectrum of cyanidin-caffeoyl-sophoroside-glucoside overlaid on its MS/MS and UV spectra adjacent to its chemical structure.

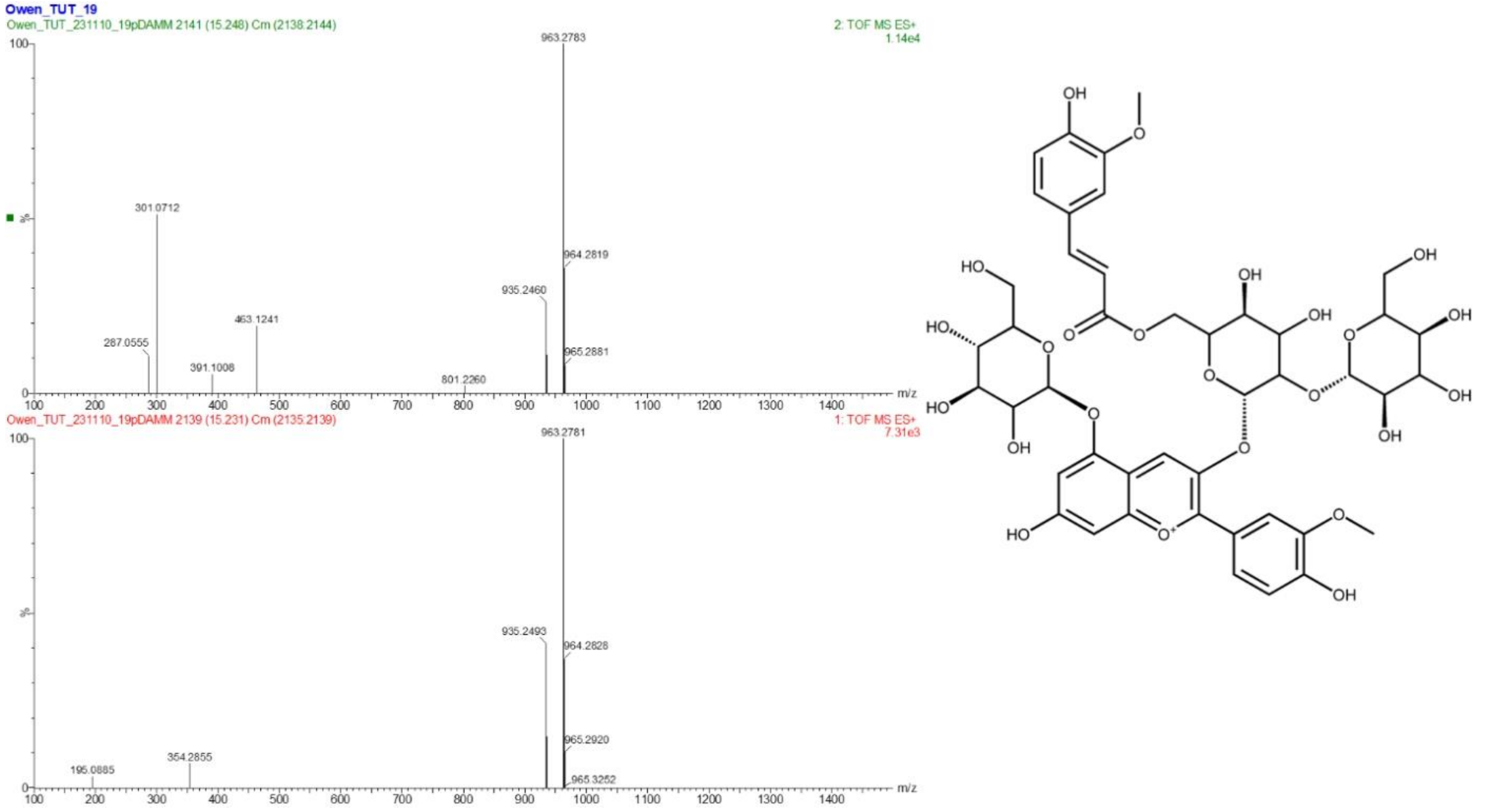

Figure S13: MS spectrum of peonidin feruloyl-sophoroside-glucoside overlaid on its MS/MS spectrum adjacent to its chemical structure.

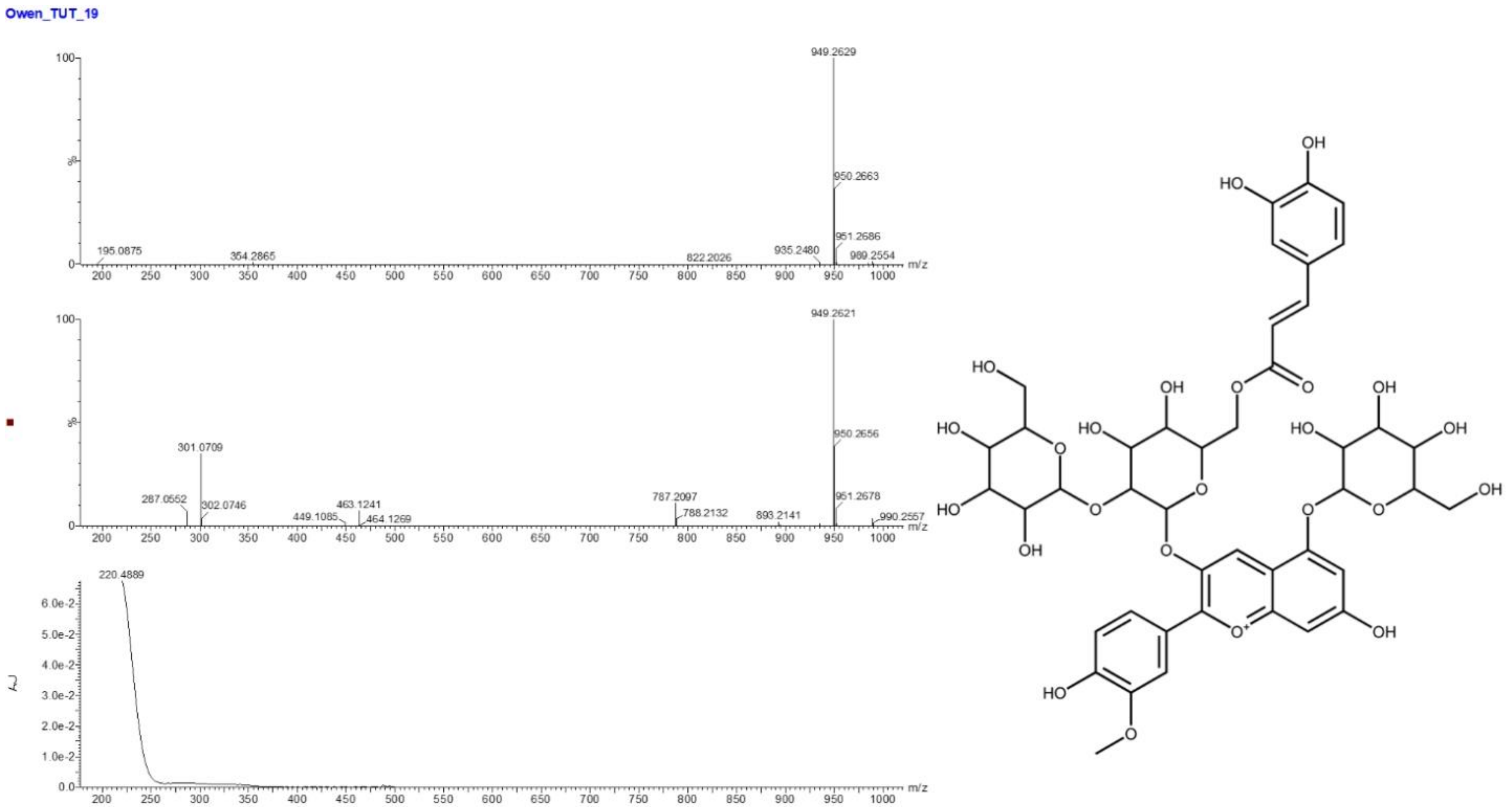

Figure S14: MS spectrum of peonidin caffeoyl-sophoroside-glucoside overlaid on its MS/MS and UV spectra adjacent to its chemical structure.

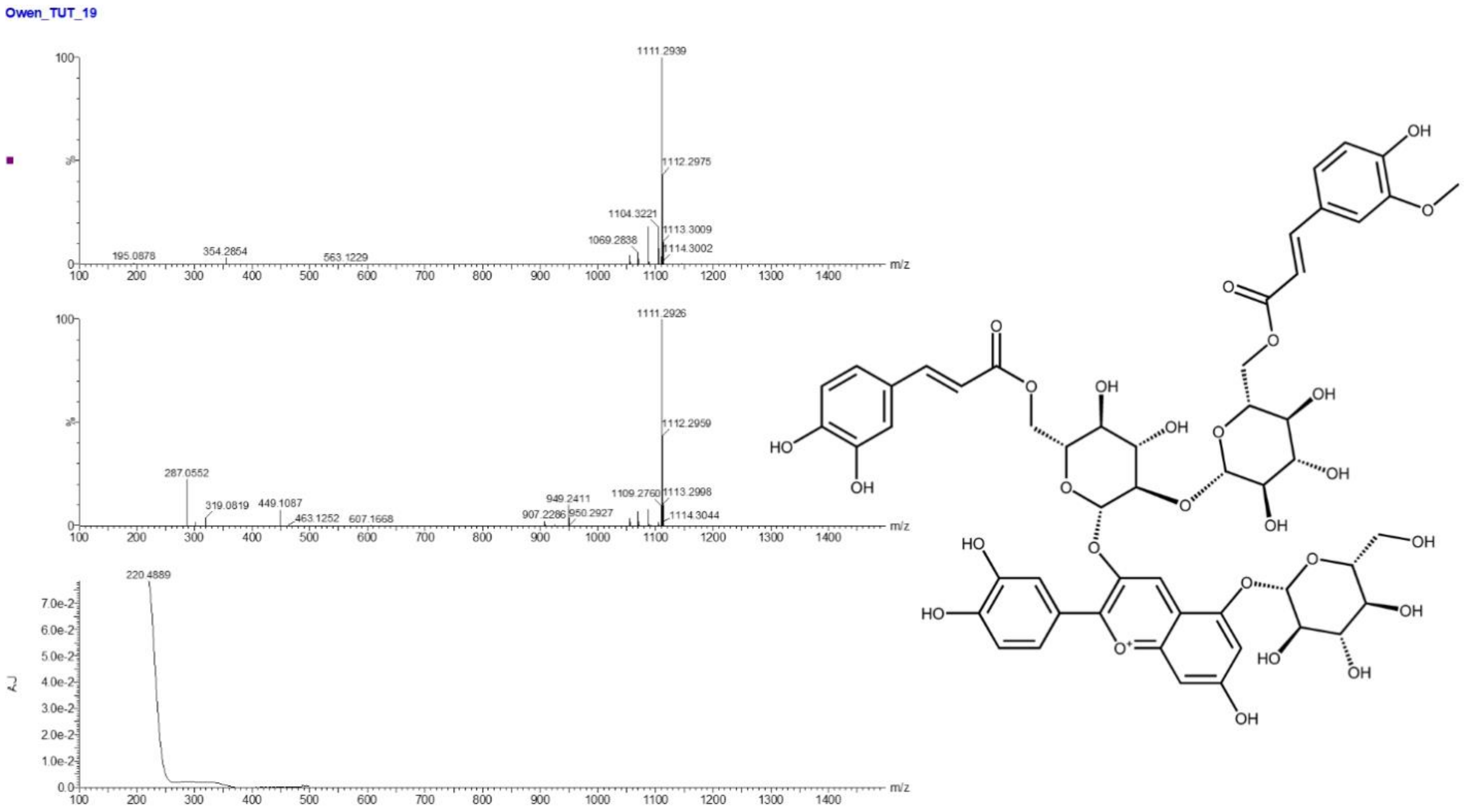

Figure S15: MS spectrum of cyanidin-caffeoyl-feruloyl-sophoroside-glucoside overlaid on its MS/MS and UV spectra adjacent to its chemical structure.

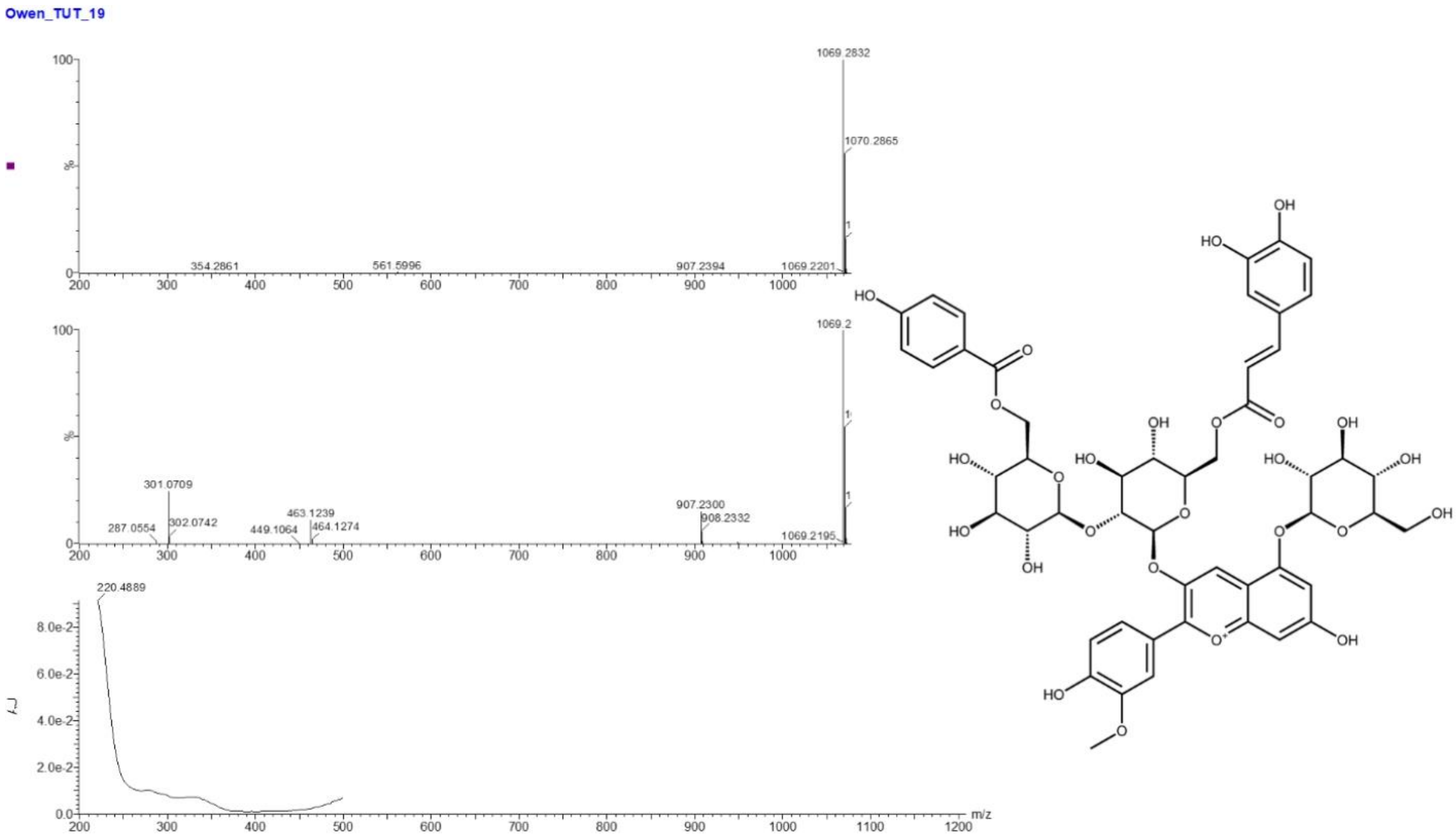

Figure S16: MS spectrum of peonidin-caffeoyl-hydroxybenzoyl-sophoroside-glucoside overlaid on its MS/MS and UV spectra adjacent to its chemical structure.

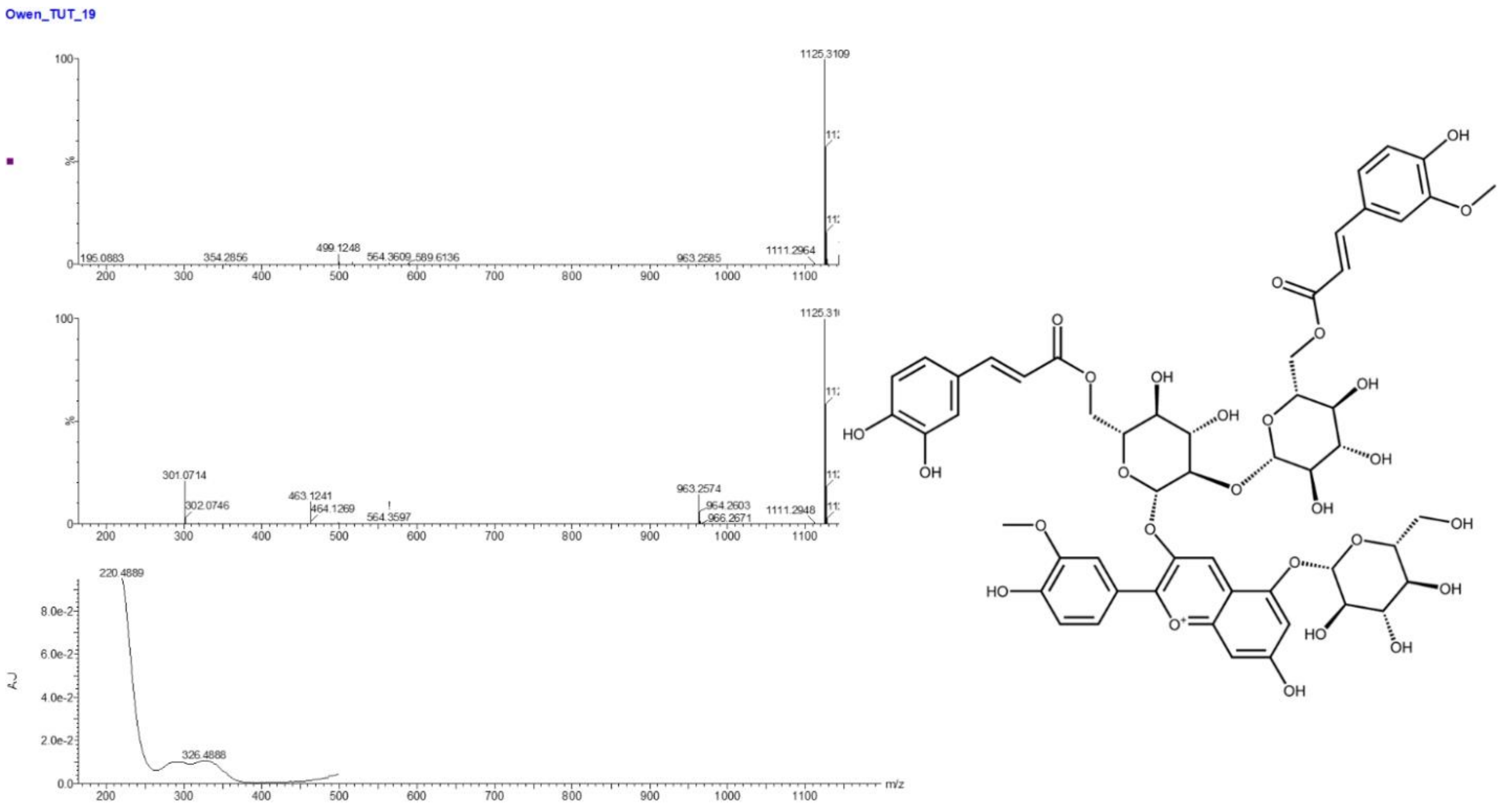

Figure S17: MS spectrum of peonidin caffeoyl-feruloyl-sophoroside-glucoside overlaid on its MS/MS and UV spectra adjacent to its chemical structure.
